# Long term efficacy and safety profile of dexamethasone intravitreal implant in retinal vein occlusions: a systematic review

**DOI:** 10.3389/fmed.2024.1454591

**Published:** 2024-11-29

**Authors:** Adriano Carnevali, Daniela Bacherini, Cristian Metrangolo, Flavia Chiosi, Pasquale Viggiano, Carlo Astarita, Valentina Gallinaro, Vincenza M. E. Bonfiglio

**Affiliations:** ^1^Department of Surgical and Medical Sciences, University Magna Graecia of Catanzaro, Catanzaro, Italy; ^2^Department of Neurosciences, Psychology, Drug Research and Child Health (NEUROFARBA), University of Florence, Florence, Italy; ^3^SC Oculistica, Ospedale di Circolo e Fondazione Macchi, Varese, Italy; ^4^Department of Ophthalmology, AORN dei Colli, Monaldi Hospital, Naples, Italy; ^5^Medical Retina and Imaging Unit, Department of Translational Biomedicine Neuroscience, University of Bari “Aldo Moro”, Bari, Italy; ^6^AbbVie S.r.l., Rome, Italy; ^7^Department of Biomedicine, Neuroscience and Advanced Diagnostic, Eye Clinic, University of Palermo, Palermo, Italy

**Keywords:** retinal vein occlusion, branch retinal vein occlusion, central retinal vein occlusion, dexamethasone intravitreal implant, macular edema

## Abstract

**Background/objective:**

Retinal vein occlusion (RVO) is a common, sight-threatening vascular disorder affecting individuals of all ages, with incidence increasing with age. Due to its complex, multifactorial nature, treating RVO remains a clinical challenge. Currently, treatment strategies include laser photocoagulation (especially for branch RVO), anti-VEGF therapies, and intravitreal corticosteroids. This systematic review (without meta-analysis) aimed to update the evidence on the efficacy and safety of the sustained-release intravitreal dexamethasone implant (DEX-i) in managing macular edema (ME) secondary to central and branch RVO.

**Methods:**

A systematic review was conducted to assess current literature on DEX-i for ME secondary to RVO. Relevant studies were analyzed for outcomes related to visual acuity, retinal thickness, and the safety profile of DEX-i in RVO treatment.

**Results:**

Evidence indicates that DEX-i substantially improves best-corrected visual acuity (BCVA) and reduces central retinal thickness (CRT) in ME associated with both branch and central RVO, demonstrating rapid and sustained effects. Common adverse events associated with DEX-i included manageable complications, such as medically controlled intraocular pressure elevation and progression of cataracts.

**Conclusion:**

DEX-i offers effective and sustained improvements in both visual and anatomical outcomes for patients with ME secondary to RVO. Individualized treatment selection is essential to optimize patient outcomes. Future directions include identifying predictive biomarkers and adopting patient-centered approaches based on individual clinical characteristics, which may enhance treatment success in RVO.

## Introduction

Retinal vein occlusion (RVO) is sight threatening vascular condition which can affect people at any age with an incidence that increases with the age (1). RVO has been traditionally subdivided into 2 main types: (1) central (CRVO), (2) branch (BRVO), and hemiretinal vein occlusion (HRVO). Although the exact cause of Central RVO (CRVO) is still unknown; the more likely hypothesis seems to be due to a consequence of a block of the central retinal vein at or proximal to the lamina cribrosa of the optic nerve (2, 3). Whereas, Branch RVO (BRVO) typically arises from compression of a tributary vein at an arteriovenous intersection (2, 3). In addition to neovascularization and neovascular glaucoma, retinal hemorrhage and macular edema (ME) may complicate either form of RVO, resulting in partial or complete loss of vision (2, 3).

It has been suggested that RVO represents the second most common retinal vascular disorder following diabetic retinopathy (4, 5). However, as compared to diabetic retinopathy, there has been relatively less interest in investigating the epidemiology of retinal vein occlusion (RVO), may be due to its lower incidence rates.

To the best of our knowledge, the first attempt to estimate the worldwide prevalence of RVO was the “Global RVO Study 2010,” published in 2010 (5). Such study estimated that there were approximately 16.4 million people with RVO (2.5 million with CRVO and 13.9 million with BRVO) (5). In 2015, the global prevalence of any RVO, BRVO and CRVO in people aged 30–89 years was 0.77, 0.64, and 0.13%, which is equivalent to an overall of 28.06 million, 23.38 million and 4.67 million affected people, respectively (6). The EURETINA White book reported that over 1.1 million individuals aged 55 and older in the European Union were affected by RVO. Of these cases, 15–25% were attributed to CRVO, while 75–80% were due to BRVO. This reflects a prevalence of RVO in either eye of approximately 0.7%. However, the available data were insufficient to provide prevalence estimates for individual countries (7).

Due to the global aging population and the subsequent increasing burden of cardiovascular diseases, it is expected that RVO might place an increasing burden on society (6, 7).

The onset of RVO has been associated with different risk factors, including advanced age, systemic hypertension, diabetes mellitus, obesity, hyperlipidemia, different cardio-vascular disorders (i.e., atherosclerosis, ischemic heart disease, etc.), smoking habit, hyperhomocysteinemia, coronavirus disease (COVID)-2019 infection, and glaucoma (3, 8–13).

Despite the close relationship between these risk factors and the onset of RVO, the pathogenesis of RVO has not been completely understood yet. As afore mentioned, ME is a common and serious complication of RVO (2, 3). Two of the most important pathogenic mechanisms of ME are the increased release of vascular endothelial growth factor (VEGF) and the production of pro-inflammatory cytokines (11). Moreover, current evidence suggests that the presence of different cytokines and chemokines in the vitreous are correlated with the occurrence of RVO, particularly the interleukin family, matrix metalloproteinases (MMP), lysophosphatidic acid-autotaxin (LPA-ATX), and platelet-derived growth factor (PDGF) (11).

Due mainly to multifactorial nature, treatment of RVO is still considered as a clinical challenge. Different strategies are currently used for treating RVO, including laser photocoagulation (for BRVO or as rescue therapy), VEGF inhibitors (anti-VEGF), and intravitreal corticosteroids (14). However, since there is currently no therapy which can solve the primary cause of RVO, all the therapeutic strategies are directed to improve the RVO complications.

### Macular edema secondary to retinal vein occlusion

While macular edema secondary to RVO (ME-RVO) is a multifactorial process driven by complex mechanisms, it is primarily attributed to an imbalance between fluid entry, fluid exit, and retinal vascular permeability (15).

VEGF has been identified as an important factor in the development of ME-RVO. Ischemic retina releases VEGF, which underlies neovascular complications, but also causes excessive vascular permeability (16, 17). The VEGF family comprises several isoforms, including VEGF-A, -B, -C, -D, -E, and placental growth factor (PGF). Notably, the overexpression of VEGF-A and PGF has been linked to an increase in vascular permeability (15, 16).

There is evidence indicating elevated levels of VEGF-A and PGF in patients with ME-RVO (15).

Increasing evidence implicates inflammation as a critical factor to the development of a wide array of retinal vascular diseases, including RVO (18). Additionally, inflammatory process may underlie many of the functional retinal vasculature alterations observed in eyes with ME-RVO (18). During inflammation a variety of soluble factors are secreted into the vitreous cavity and their concentrations increases in eye with ME-RVO, either secondary to BRVO (ME-BRVO) or to CRVO (ME-CRVO) (19–21) and may affect visual prognosis (18).

**FIGURE 1:**
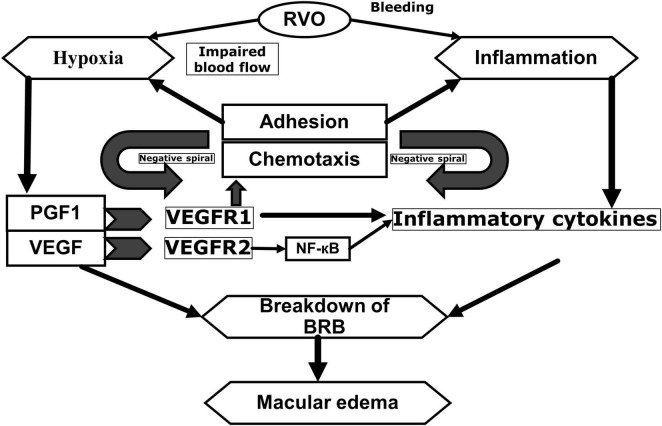
Pathophysiology of macular edema secondary to retinal vein occlusions. Adapted from Noma et al. (20) and Noma et al. (21). RVO, retinal vein occlusion; PGF1, placental growth factor; VEGF, vascular endothelial growth factor; NF-êB, nuclear factor kappa light chain enhancer of activated B cells; BRB, blood-retinal barrier; VEGF R1, vascular endothelial growth factor receptor 1; VEGF R2, Vascular endothelial growth factor receptor 2.

These inflammatory cytokines may be responsible of the transition from an acute to chronic inflammation, which causes increase in vascular permeability and the development of ocular neovascularization (18–21). In other words, in eyes with RVO, ME develops because of reduced venous drainage and increased capillary leakage/permeability, which is increased by a variety of pro-angiogenic and inflammatory mediators (Figure 1).

Given that the two primary mechanisms underlying ME secondary to RVO (ME-RVO) involve increased VEGF release and the production of pro-inflammatory cytokines, the main treatment options for ME-RVO include intravitreal injections of anti-VEGF agents and sustained-release intravitreal corticosteroid implants (14, 22).

Moreover, the relevance of the inflammatory pathway on the onset of ME-RVO has been clearly suggested by the currently available scientific evidence that shows the effectiveness of intravitreal steroids treatments in patients with RVO (22–25).

The current systematic review aimed to provide an updated summary of the effectiveness and safety of the sustained release intravitreal dexamethasone implant (DEX-i) for the treatment of ME secondary to either CRVO or BRVO.

### Pivotal trial: the lessons from the GENEVA study

GENEVA study was a RCT that evaluated the efficacy and safety of DEX-i in patients with ME-RVO (either BRVO or C RVO) (23, 25). The results of the GENEVA study showed that, as compared to the control group, the eyes treated with DEX-i achieve a BCVA improvement ≥ 15 letters in less time (*p* < 0.001); a greater proportion of eyes achieved a BCVA improvement ≥ 15 letters at days 30 to 90 (*P* < 0.001); while the proportion of eyes with a ≥ 15-letter loss in BCVA was significantly lower at all follow-up visits (*P* ≤ 0.036). In addition, CRT was significantly reduced at all the point-measured in the eyes treated with DEX-i (23, 25).

## Materials and methods

### Search strategy and eligibility criteria

PubMed, Medline, Embase, and Google Scholar databases were examined to identify randomized controlled trials (RCT) and real-world evidence (RWE) studies that assessed the central retinal thickness (CRT); and/or central subfoveal thickness (CSF); and/or central macular thickness (CMT); and/or best corrected visual acuity (BCVA) from January 2018 to September 2023.

The search strategy for the outcome was performed for mesh terms “Retinal vein occlusion” AND “Macular edema” AND “Dexamethasone intravitreal implant” OR “Ozurdex” OR “Vascular endothelial growth factor.” In addition, as free search in title and abstract using a series of key words have been included, such as retinal vein occlusion, branch retinal vein occlusion, central retinal vein occlusion, ischemic retinal vein occlusion, nonischemic retinal vein occlusion, hemiretinal vein occlusion, retinal vein thrombosis, and retinal venous thrombosis AND macular edema AND treatment.

The current study included RCT and retrospective and prospective RWE studies conducted on patients with ME-RVO who underwent treatment with DEX-i 0.7 mg or anti-VEGF and for which the final CRT or change in CRT and/or final BCVA or change in BCVA from baseline were reported. Letters and case series with less than 10 subjects were excluded.

Studies that did not include CRT/BCVA outcomes following DEX-i; studies published in other language different from English, French, Portuguese, Italian, or Spanish (Those studies written in other languages, but with an abstract available in English with sufficient information, were also included); and conditions other than ME-RVO were excluded.

We reviewed references by evaluating their titles and abstracts, and selected studies that were relevant for further analysis. We manually checked the reference lists of relevant studies, systematic reviews, and meta-analyses to find any additional publications that could be useful for our analysis.

The Preferred Reporting Items for Systematic Reviews and Meta-Analyses (PRISMA^1^) statement was developed to facilitate improved reporting of systematic review (26, 27). However, it is not free of limitations, since it provides limited guidance on reporting certain aspects of the review, such as the methods for presentation and synthesis (26–29). In addition, the high level of heterogeneity among the different studies makes difficult, if not impossible, to combine the data (27). Moreover, narrative reviews have serious shortcomings, including lack of transparency, methods deficiencies, or inadequate reporting of the review limitations (30, 31).

Synthesis Without Meta-analysis (SWiM) has emerged as systematic review to address questions to which meta-analysis may not be able to provide an adequate answer (29). Indeed, approximately 1/3 of health-related systematic reviews of interventions do not include meta-analysis (29).

This systematic review without meta-analysis was carried out following the guidelines outlined in the PRISMA statement (32) (See Supplementary Table 1).

### Study selection and data extraction

The authors independently generated the queries for the literature search and selected the articles fulfilling the criteria established for each subject and solved any disagreement through discussion and consensus. To determine the eligibility, searched papers, including title, abstract, and full text were evaluated.

The data that were extracted included information about the study (first author, publication time, length of the follow-up, RVO subtype, and study type); therapy information; and efficacy parameters (retinal thickness and/or BCVA).

Other research data, including number of intravitreal injections and/or incidence of adverse events were also assessed.

## Results

### Search results and study characteristics

**FIGURE 2:**
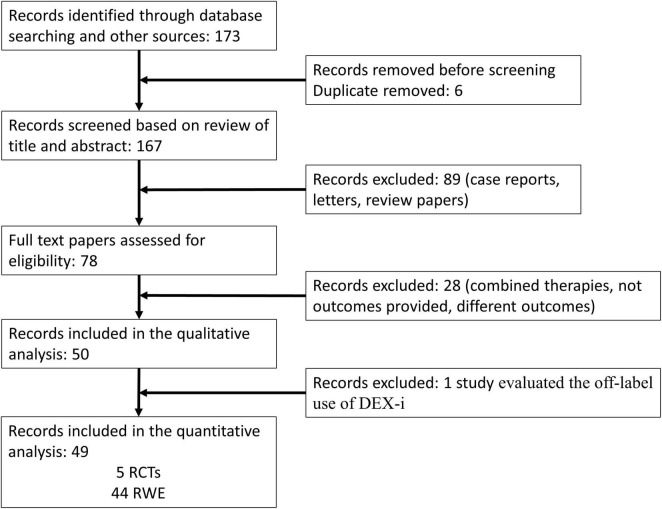
PRISMA 2009 flow diagram. DEX-i, dexamethasone intravitreal implant; RCTs, randomized controlled trials; RWE, real-world evidence.

The flow chart of the selection process is shown in Figure 2. A total of 173 articles were identified through database searching. After removing duplicates, 167 articles were evaluated by their titles and abstracts. Out of these, 78 studies met the criteria for a full-text review. Among the remaining 78 papers that underwent a full review, 28 were excluded due to incomplete data, assessment of combined therapy, and/or different outcomes. Finally, a total of 50 papers, 5 RCT and 45 RWE studies, were eligible for the qualitative analysis. Of these 50 studies, Yilmaz et al. (33) evaluated the off-label use of DEX-i and did not provide efficacy data; therefore, this study was eliminated (Figure 2).

We have evaluated the RCT and RWE studies separately, in order to present the data as accurately as possible.

### Randomized control trails

Five RCTs met the inclusion/exclusion criteria and were included in the study (34–38). Among them, three studies (34, 35, 38) reported data at month-6 and two (36, 37) reported data at month-12.

All the RCTs demonstrated significant CRT reduction and BCVA improvement after DEX-i treatment, both in eyes with BRVO and in those with CRVO (34–38).

The Table 1 summarizes the main anatomic and visual outcomes of DEX-I according to the RCTs included in the study.

**TABLE 1:** An overview of the main results from randomized clinical trials with the intravitreal dexamethasone implant (DEX-i) included in the current study.

Study	References	Eyes (n)	Disease	LOFU, months	Mean number of injections	Mean BCVA difference (letters or logMAR) between baseline and at end of the follow-up	Mean CRT (μ m) change from baseline at end of follow-up	Maximum BCVA change (letters or logMAR)	Maximum CRT change (μ m)
Li et al.	(34)	63 66	BRVO CRVO	6	N.A. N.A.	N.A. N.A.	N.A. N.A.	+11.4 ± 9.6 +9.8 ± 11.0	-323 ± 189^1^ -487 ± 203^1^
COMRADE-B	(35)	118	BRVO	6	1.0 ± 0.0	+9.2 ± 12.5	-112.3 ± 172.1	N.A.	N.A.
COMO	(36)	154	BRVO	12	2.5	+7.4 ± 1.0*	−227*,^†^	+10.0 ± 0.6*	N.A.
COMRADE**	(37)	40 22	BRVO CRVO	12	0.40 ± 0.50^ 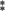 ^ 0.45 ± 0.51^ 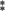 ^	+12.3 ± 13.4 +13.5 ± 21.3	−211.5 ± 199.3 −360.3 ± 260.2	+14.3 ± 11.9 +15.7 ± 20.5	−265.4 ± 198.5 −445.4 ± 249.8
Kumar et al.	(38)	15	BRVO	6	1.0 ± 0.0	+9.50 ± 9.60	−205.66 ± 136.6	+16.00 ± 9.30	−268.27 ± 85.72

^1^Results at month-2.

*Least-squares mean difference ± standard error.

^†^It does not provide data about standard error.

**COMRADE Extension.

^
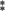
^Patients received 1.00 ± 0.00 DEX-i during the COMRADE-B and COMRADE-C core studies (References 34,38). LOFU, length of follow-up; BCVA, best corrected visual acuity; CRT, central retinal thickness; BRVO, branch retinal vein occlusion; CRVO, central retinal vein occlusion; DEX-i, dexamethasone intravitreal implant; ETDRS, Early Treatment Diabetic Retinopathy Study; NA, not available.

### Changes in central retinal thickness

Changes in retinal thickness has been considered as an anatomical outcome to evaluate the efficacy of ME treatment.

#### Branch retinal vein occlusion

Five studies evaluated the anatomic and visual outcomes in eyes with BRVO (34–38).

Li et al. (34) specifically examined the mean central retinal thickness change at month 2. This study identified a substantial CRT change of −323 ± 189 μm in eyes affected by macular edema secondary to BRVO (*p* < 0.001) (34).

Among the RCTs that reported anatomic outcomes at the endpoint, the mean CRT reduction ranged between -112.3 ± 172.1 μm (COMRADE-B, Reference 32) and –211.5 ± 199.3 μm (COMRADE Extension, Reference 36).

According to the results of these RCTs, CRT was reduced significantly after DEX-i administration.

#### Central retinal vein occlusion

Two RCTs (34, 37) investigated the impact of intravitreal dexamethasone implant in eyes affected by macular edema secondary to CRVO. According to the findings of these RCTs, central retinal thickness (CRT) showed a significant reduction after DEX-i administration, with changes reported as −487 ± 203 μm (28) and by -445.4 ± 249.8 μm (37).

Although COMRADE C is outside the inclusion criteria window of this publication, we briefly discuss its main findings. This paper compared the efficacy and safety of DEX-i and intravitreal injections of ranibizumab in patients with ME-CRVO (39). According to the COMRADE C results, one DEX-i significantly increased mean BCVA at months 1 and 2, with no significant differences versus ranibizumab. Although at month-6 one DEX-i increased mean BCVA by 2.96 letters, this rise was less than that observed with ranibizumab. This seems to be due to a single injection of DEX-i, while eyes treated with ranibizumab continued to receive repeated intravitreal injections (39).

### Changes in best-corrected visual acuity

Improvements in BCVA have been frequently used to evaluate treatment efficacy.

#### Branch retinal vein occlusion

In a similar way to CRT, Li et al. (34) reported data at month-2, reporting a mean BCVA improvement of +11.4 ± 9.6 ETDRS letters after DEX-i administration. Additionally, these authors reported that 34.9% of eyes achieved ≥ 15-letter BCVA improvement from baseline (34).

The COMO study reported the changes of BCVA as least-squares mean difference ± standard error instead of as mean ± standard deviation (36). They found a BCVA gain of +7.4 ± 1.0 letters at month-12 (36).

At the study endpoint, the mean BCVA improvement ranged from +9.2 ± 12.5 letters of the COMRADE-B (35) and +12.3 ± 13.4 letters of the COMRADE Extension (37).

#### Central retinal vein occlusion

Mean BCVA was significantly improved by +9.8 ± 11.0 letters (34) and +13.5 ± 21.3 letters (37) after DEX-i injection.

### Real-word evidence

Between January 2018 and September 2023, we have identified 44 RWE studies that evaluated the visual and/or anatomic and/or safety outcomes in patients with either BRVO and/or CRVO (40–83) (Table 2). Among them, two studies (56, 83) assessed only safety outcomes.

**TABLE 2:** An overview of the main results from the real-world studies with the intravitreal dexamethasone implant (DEX-i) included in the current study.

Study	References	Design	Eyes (n)	Disease	LOFU, months	Mean number of injections	Mean BCVA difference (letters or logMAR) between baseline and at end of the follow-up	Mean CRT (μm) change from baseline at end of follow-up	Maximum BCVA change (letters or logMAR)	Maximum CRT change (μm)
Winterhalter et al.	(40)	R	31 17	BRVO CRVO	6	1.13 ± 0.34 1.0 ± 0.0	−0.22 ± 0.26^a^ +0.09 ± 0.39^a^	309 (275–487)^b,c^ 409 (291–704)^b,c^	−0.23 ± 0.25^a^ −0.02 ± 0.3^a^	279 (257–308)^b^ 306 (254–569)^b^
Altunel et al.	(41)	P	73	BRVO	2	1.0 ± 0.0	0.54 ± 0.20^a^	−334.5 ± 99.1^a^	N.P.	N.P.
Hussain et al.	(42)	R	10 12	BRVO CRVO	6	1.9 ± 0.9 2.5 ± 0.7	0.11 ± 0.17^a^ −0.22 ± 0.41^a^	−78.0 ± 110.8^a^ −163.0 ± 156.6^a^	−0.12 ± 0.19^a^ −0.18 ± 0.31^a^	−169.0 ± 90.0^a^ −252.0 ± 164.5^a^
Yuksel et al.	(43)	R	15	BRVO	6	2.4 ± 1.5	−0.27 ± 0.53^a^	−166.4 ± 130.2^a^	−0.27 ± 0.53^a^	−166.4 ± 130.2^a^
Lip et al.^1^	(44)	R	28 9 29	BRVO HRVO CRVO	12	1.1 ± 0.8	0.54 (0.30–0.78)^1,b^ 0.44 (0.15–0.54)^1,b^ 0.48 (0.18–0.78)^1,b^	252 (224–347)^1,b^ 285 (243–386)^1,b^ 236 (208–332)^1,b^	N.P.	N.P.
Bulut et al.	(45)	P	15	CRVO	6	1.0 ± 0.0	−0.48 ± 0.70^a^	−67.0 ± 132.5^a^	−0.48 ± 0.67^a^	−94.8 ± 122.4^a^
Mishra et al.	(46)	P	20	CRVO	6	1.1 ± 0.3^a^	−0.42 ± 0.39^a^	−314.0 ± 19.2^a^	N.P.	N.P.
Yoon et al.	(47)	P	71	BRVO	12	2.2 ± 0.9^a^	+15.3 ± 15.0	−196.9 ± 164.1	+18.6 ± 12.9	−246.8 ± 150.7
Garay-Aramburu et al.	(48)	R	10	BRVO CRVO	60^d^	4	−0.36 ± 0.23^a^	−249.6 ± 178.7^a^	N.P.	N.P.
Simsek et al.	(49)	P	40 31	BRVO CRVO	4	1.0 ± 0.0	−0.15 ± 0.66^a^ −0.37 ± 0.68^a^	−135.9 ± 166.7^a^ −278.4 ± 1,809.4^a^	−0.34 ± 0.63^a^ −0.56 ± 0.64^a^	−258.9 ± 153.9^a^ −387.9 ± 169.7^a^
Blanc et al.^1^	(50)	R	21 29	BRVO CRVO	36	5.0 (1.0–1.0)	60.0 (30.0–75.0)^1,b^	285.0 (231.0–331.0)^1,b^	+10.0 (0.0–20.0)^1^	206.0 (197.0–228.0)^1,b^
Kaldırım et al.	(51)	R	20	BRVO	6	1.0 ± 0.0	−0.26 ± 0.17^a^	−163.2 ± 78.5^a^	−0.41 ± 0.12^a^	−253.6 ± 64.6^a^
Donati et al.	(52)	P	8 18	BRVO CRVO	6	1.0 ± 0.0	−0.22 ± 0.37^a^	−103.3 ± 160.4^a^	−0.22 ± 0.37^a^	−175.6 ± 145.0^a^
Niro et al.	(53)	P	15	CRVO	12	1.3 ± 0.5^a^	0.41 ± 0.36	−282.8 ± 109.6	0.41 ± 0.36	−282.8 ± 109.6
Houben et al.	(54)	R	32 40	BRVO CRVO	6	1.0 ± 0.0	−0.01 ± 0.19 0.03 ± 0.49	−122.5 ± 152.5 −202.3 ± 194.1	−0.06 ± 0.19 −0.15 ± 0.39	−146.4 ± 142.3 −202.3 ± 194.1
Ozkaya et al.	(55)	R	41	BRVO	24	2.7 ± 1.1	−0.06 ± 0.59^a^	−256.0 ± 106.2^a^	−0.16 ± 0.56^a^	−256.0 ± 106.2^a^
Tufail et al.^2,h^	(56)	P	385 257	BRVO CRVO	24	2.2 ± 1.3	N.P.	N.P.	N.P.	N.P.
Yucel et al.	(57)	R	24	CRVO	6	1.62 ± 0.5	−0.11 ± 0.56^a^	−193.9 ± 219.4^a^	−0.21 ± 0.50^a^	−348.1 ± 171.8^a^
Eris et al.	(58)	R	52	BRVO	6	1.0 ± 0.0	0.32 ± 0.79^a^	−78.0 ± 183.1^a^	−0.18 ± 0.69^a^	−280.0 ± 155.6^a^
Georgalas et al.	(59)	P	13 10	BRVO CRVO	12	2.0 ± 0.0	−0.26 ± 0.15^a^ −0.11 ± 0.26^a^	−187.1 ± 137.8^a^ −119.8 ± 197.3^a^	−0.33 ± 0.10^a^ −0.52 ± 0.23^a^	−302.9 ± 112.9^a^ −408.1 ± 167.3^a^
Teja et al.	(60)	R	28	RVO	6	2.7 ± 2.3	−0.31 ± 0.68^a^	−68.0 ± 153.9^a^	−0.45 ± 0.67^a^	−160.7 ± 39.6^a^
Bayat et al.	(61)	R	19	BRVO	6	1.75 ± 0.44	−0.61 ± 0.39^a^	−247.0 ± 73.3^a^	−0.61 ± 0.39^a^	−247.0 ± 73.3^a^
Dikel et al.	(62)	R	16	BRVO	12	1.9 ± 0.6^a^	−0.21 ± 0.59^a^	−150.9 ± 95.1^a^	−0.28 ± 0.60^a^	−203.4 ± 80.9^a^
Nikula et al.	(63)	R	12 24	BRVO CRVO	48 W	1.4 ± 0.5^a^ 1.3 ± 0.5^a^	−0.21 ± 0.23^a^ −0.41 ± 0.20^a^	−179.5 ± 82.9^a^ −249.4 ± 95.2^a^	−0.21 ± 0.23^a^ −0.41 ± 0.20^a^	−179.5 ± 82.9^a^ −249.4 ± 95.2^a^
Sever et al.	(64)	R	18 17	BRVO MVO	12	2.0 ± 0.0 1.3 ± 0.4	−0.11 ± 0.20 −0.48 ± 0.08	−47.4 ± 31.9 −40.6 ± 23.2	N.P. −0.48 ± 0.08	N.P. N.P.
Wallsh et al.	(65)	R	90 59	BRVO CRVO	10 y^e^	3.1 4.1	−0.10 ± 0.06^3,a^ −0.09 ± 0.08^3,a^	−61.7 ± 11.8^3,a^ −47.1 ± 14.1^3,a^	N.P. N.P.	N.P. N.P.
Erogul et al.	(66)	R	22 19	BRVO CRVO	3	N.P.	N.P.	−269.0 ± 169.8^a^	N.P.	N.P.
Arrigo et al.	(67)	R	135 178	BRVO CRVO	7 y^f^	9.80 ± 5.39 10.70 ± 4.76	−0.23 ± 0.42^a^ −0.16 ± 0.25^a^	−137.7 ± 196.6^a^ −137.7 ± 176.1^a^	N.P. N.P.	N.P. N.P.
de Salles et al.	(68)	R	35 31	BRVO CRVO	5 y^g^	8.6 ± 0.7^a^ 10.3 ± 0.9^a^	−2.1 ± 23.4^a^ −9.7 ± 32.6^a^	−140.0 ± 173.5^a^ −185.0 ± 266.7^a^	N.P. N.P.	N.P. N.P.
Huang et al.	(69)	R	29	BRVO	30	N.P.^i^	+19.8 ± 24.4	−154.0 ± 133.5^a^	+29.5 ± 23.5	−209.1 ± 125.1^a^
Wecker et al.	(70)	R	99	BRVO CRVO	36	2.2 ± 2.1	−1.8 ± 23.0	−95.0 ± 189.3^a^	N.P.	−95.0 ± 189.3^a^
Harb et al.	(71)	P	38	BRVO CRVO	12	1.6 ± 0.9	N.P.	N.P.	0.22 ± 0.17	−248.1 ± 159.7
Kim et al.	(72)	P	554 146	BRVO CRVO	6	1.1 1.2	−0.11 ± 0.39^a^ −0.12 ± 0.40^a^	N.P. N.P.	−0.17 ± 0.37^a^ −0.18 ± 0.41^a^	N.P. N.P.
Garay-Aramburu et al.^1^	(73)	R	77 34	BRVO CRVO	44	3 ± 4^1^	5 ± 15^1^	−184.5 ± 211^1^	N.P.	N.P.
Castro-Navarro et al.	(74)	R	42 15	BRVO CRVO	6	1.0 ± 0.0	+6.1 (- 2.8 to 14.9)^4^ -9.5 (-25.5 to 6.5)^4^	257.3 (-351.9 to -162.6)^4^ - 194.2 (- 340.7 to 47.7)^4^	N.P.	−299.9 ± 181.3
Jirarattana sopa et al.	(75)	R	12 2 14	BRVO HRVO CRVO	6	1.4 ± 0.5	−0.07 ± 0.40^a^	−179.3 ± 172.2^a^	−0.21 ± 0.38^a^	−258.4 ± 130.6^a^
Zhang et al.	(76)	R	6 2	BRVO CRVO	5 y	1.3 ± 0.5	−0.02 ± 0.31^a^	−377.4 ± 184.6^a^	−0.24 ± 0.21^a^	N.P.
Moreno-López et al.	(77)	P	27 17	BRVO CRVO	6	1.0 ± 0.0	−0.45 ± 0.37^a^ −0.42 ± 0.43^a^	−315.1 ± 153.5^a^ −413.0 ± 187.1^a^	N.P. N.P.	N.P. N.P.
Horozoglu et al.	(78)	R	18 10	BRVO CRVO	3	1.0 ± 0.0	N.P:	195.1 ± 22.5^a^	N.P.	−260.3 ± 22.0^a^
Soliman et al.	(79)	R	862 688	BRVO CRVO	24	1.81 ± 0.9	−0.05 ± 0.50	N.P.	−0.23 ± 0.28	N.P.
Ozcift et al.^2^	(80)	R	23	BRVO	6	N.P.	1.00^b,j^	367.0^b,j^	0.70^b,j^	240.0 ^b,j^
Yu-Chuan Kang et al.^a^	(81)	R	49	CRVO	12	N.P.	−0.29 (−0.49 to −0.09)*^,^^1^ −0.50 (−0.82 to −0.17)**^,^^1^	−96.8 (−174.4 to 19.1)*^,^^1^ −96.3 (−197.2 to −4.5)**^,^^1^	−0.33 (−0.52 to −0.13)*^,^^1^ −0.50 (−0.82 to −0.17)**^,^^1^	−96.8 (−174.4 to 19.1)*^,^^1^ −110.7 (−204.3 to −17.2)**^,^^1^
Darabuş et al.	(82)	P	15	BRVO	6	1.0 ± 0.0	−0.36 ± 0.30^a^	−127.0 ± 88.0^a^	−0.36 ± 0.30^a^	−127.0 ± 88.0^a^
Ayaz et al.^2,h^	(83)	R	91 45	BRVO CRVO	24	N.P.	N.P.	N.P.	N.P.	N.P.

BCVA improvements are shown in negative values with LogMAR and in positive values in ETDRS letters.

^a^CRT and BCVA data were calculated from the study.

^b^The study provided absolute values instead of changes from baseline.

^c^Mean difference (95% confidence interval) between baseline and follow-up on log scale: 0.21 (0.08 to 0.34); *p* = 0.001 (BRVO) and 0.23 (0.06 to 0.40); *p* = 0.008 (CRVO).

^d^Mean follow-up: 65.5 months; range: 60.7–68.3 months.

^e^Up to 10 years of follow-up. Efficacy data refers to “last visit”

^f^Mean follow-up was 45 ± 25 months (range 12–84 months).

^g^Mean time of follow-up was 24.7 ± 18.6 and 25.4 ± 19.7 months in the BRVO and CRVO groups, respectively.

^h^The study provided information only about adverse events.

^i^Thirteen eyes received one DEX-i and 16 eyes received multiple DEX-i.

^j^Median (interquartile range) baseline BCVA = 1.1 (0.52–3.10) logMAR. Median (interquartile range) baseline CRT = 515.00 (318–770) μm.

^1^Median (interquartile range).

^2^The study did not provide efficacy data.

^3^Mean ± standard error of the mean.

^4^CRT and BCVA are expressed as mean (95% confidence interval).

*Eyes eligible for pRCTs.

**Eyes ineligible for pRCTs. R, retrospective; P, prospective; CRT, central retinal thickness; BCVA, best corrected visual acuity; RVO, retinal vein occlusion; BRVO, branch retinal vein occlusion; CRVO, central retinal vein occlusion; HRVO, Hemi-retinal vein occlusion; MVO, macular vein occlusion; DEX-i, dexamethasone intravitreal implant; ETDRS, Early Treatment Diabetic Retinopathy Study; NP, not provided; pRCTs, pivotal randomized control trials.

In addition, two studies (44, 75) included patients with Hemi-retinal vein occlusion (9 and 2 eyes, respectively).

Although most studies were conducted on previously treated patients, 8 (49, 50, 63, 66, 69, 71, 72, 74) RWE studies assessed treatment naïve eyes, which will be analyzed individually in the next section.

A Registry database, which included information from 16 National Health Service hospitals, assessed the efficacy and safety of DEX-i in 688 eyes with CRVO and 862 eyes with BRVO (79). Of the total sample, 1,250 eyes (80.6%) were naive (although the paper did not provide detailed information on that group). In the overall study sample, mean VA was significantly increased by 0.23 ± 0.38 (*p* < 0.001). In particular, 1,271 eyes (82%) received ≤ 2 DEX-i throughout the 2 years of study follow-up. The median time between the first and the second DEX-i was 108 days (79).

According to the results of a systematic review and meta-analysis that included 12 RWEs (84), CRT was significantly reduced after DEX-i administration in eyes with BRVO. Additionally, BCVA tended to be improved following DEX-i treatment, although not all the studies found a significant visual improvement after treatment. This problem seems to be due to a not proper injection time of DEX-i, since in some studies retreatment with DEX-i was carried out every 6 months, and also because some studies did not report the retreatment periods. Another possible explanation was the presence of cataract in some patients which was not performed throughout the study (84). Regarding the eyes with CRVO, this systematic review reported substantial CRT reduction (ranging between −137.0 and −256 μm) and visual acuity improvement after DEX-i injection (84).

### Efficacy and durability of DEX-i in treatment naïve patients

In eyes with ME-RVO, both BRVO and CRVO, starting an effective treatment at early stages has been associated with better visual outcome and fewer long-term complications (14, 22, 85–87).

Different RWE studies have evaluated the efficacy and safety of DEX-i in treatment naïve eyes with ME-RVO, either BRVO or CRVO (49, 50, 63, 66, 69, 71, 72, 74).

Yuksel et al. (43), retrospectively assessed the efficacy and safety of DEX-i in 15 eyes with ME secondary to BRVO, of which, 13 eyes were treatment-naïve. This study has not provided detailed clinical outcomes of treatment-naïve eyes, so it cannot be analyzed. Nevertheless, they provided information about the two eyes that had received previous treatment, one patient gained 10 letters and -30 μm regression on optical coherence tomography; while in the other one, ME decreased by -188 μm but vision did not improve.

Kim et al. (72), found that within the subgroups of patients diagnosed with BRVO or CRVO, those who were treatment-naïve at baseline achieved greater BCVA improvement than those previously-treated. Indeed, peak BCVA gain from baseline among eyes with BRVO was +14.4 versus +6.6 ETDRS in the treatment-naïve and non-treatment-naïve subgroups, respectively; Whereas, for the eyes affected by CRVO, BCVA improvement from baseline were +20.9 and +8.5 ETDRS letters in the treatment-naïve and non-treatment-naïve subgroups, respectively (72).

Castro-Navarro et al. (74) analyzed 31 eyes with ME-RVO, who were treatment-naïve at baseline. They observed that the BCVA improvement from baseline was significantly greater in the treatment-naïve eyes than in those previously treated (mean difference: +18.0 letters; 95% CI: +4.0 to +35.0; *p* = 0.0129). In addition, CRT reduction was also greater in the treatment-naïve subgroup, although such a difference was not statistically significant (mean difference: 41.5 μm; 95% CI: -120.0 μm to 197.0 μm; *p* = 0.6251) (74).

Gale et al. (88) performed a retrospective chart review that included data from 5,661 treatment-naive patients (collected in 27 National Health Service Trust hospitals between February 2002 and September 2017) with a single mode of treatment (i.e., a single type of treatment throughout follow-up) for ME secondary to BRVO. They included data from 676 eyes treated with DEX-i (88). Mean visual acuity was 53.1, 59.7, 57.6, 56.1, 59.3, and 62.9 ETDRS letters at baseline, and 6, 12, 18, 24, and 36 months, respectively. The mean number of DEX-i injected was 1.3, 1.5, 1.7, 1.7, and 1.7 at 6, 12, 18, 24, and 36 months, respectively. This study did not provide any anatomic outcomes (88).

Table 3 shows the main results of the 8 studies that have evaluated the effectiveness of DEX-i in treatment-naïve eyes with ME-RVO.

**TABLE 3:** An overview of the main results from the Real-world studies with the intravitreal dexamethasone implant (DEX-i) in treatment-naïve eyes with macular edema secondary to retinal vein occlusion (ME-RVO).

Study	References	Design	Eyes (n)	Disease	LOFU, months	Mean number of injections	Mean BCVA difference (letters or logMAR) between baseline and at end of the follow-up	Mean CRT (μ m) change from baseline at end of follow-up	Maximum BCVA change (letters or logMAR)	Maximum CRT change (μ m)
Simsek et al.	(49)	P	40 31	BRVO CRVO	4	1.0 ± 0.0	−0.15 ± 0.66^a^ −0.37 ± 0.68^a^	−135.9 ± 166.7^a^ −278.4 ± 1,809.4^a^	−0.34 ± 0.63^a^ −0.56 ± 0.64^a^	−258.9 ± 153.9^a^ −387.9 ± 169.7^a^
Blanc et al.^1^	(50)	R	21 29	BRVO CRVO	36	5.0 (1.0–1.0)	60.0 (30.0–75.0)^1,b^	285.0 (231.0–331.0)^1,b^	+10.0 (0.0–20.0)^1^	206.0 (197.0–228.0)^1,b^
Nikula et al.	(63)	R	12 24	BRVO CRVO	48 W	1.4 ± 0.5^a^ 1.3 ± 0.5^a^	−0.21 ± 0.23^a^ −0.41 ± 0.20^a^	−179.5 ± 82.9^a^ −249.4 ± 95.2^a^	−0.21 ± 0.23^a^ −0.41 ± 0.20^a^	−179.5 ± 82.9^a^ −249.4 ± 95.2^a^
Erogul et al.	(66)	R	22 19	BRVO CRVO	3	N.P.	N.P.	−269.0 ± 169.8^a^	N.P.	N.P.
Huang et al.	(70)	R	29	BRVO	30	N.P.^g^	+19.8 ± 24.4	−154.0 ± 133.5^a^	+29.5 ± 23.5	−209.1 ± 125.1^a^
Harb et al.	(71)	P	38	BRVO CRVO	12	1.6 ± 0.9	N.P.	N.P.	0.22 ± 0.17	−248.1 ± 159.7
Kim et al.	(72)	P	235 65	BRVO CRVO	6	N.P. N.P.	N.P. N.P	N.P. N.P.	−0.29^c^ −0.42^d^	N.P. N.P.
Castro-Navarro et al.	(74)	R	22 9	BRVO CRVO	6	1.0 ± 0.0	11.0 (0.4 to 21.6)^2^	- 269.8 (-399.4 to -140.3)^2^	N.P.	N.P.

BCVA improvements are shown in negative values with LogMAR and in positive values in ETDRS letters.

^a^CRT and BCVA data were calculated from the study.

^b^The study provided absolute values instead of changes from baseline.

^g^Thirteen eyes received one DEX-i and 16 eyes received multiple DEX-i.

^c^Approximately +14.4 ETDRS letters.

^d^Approximately +20.9 ETDRS letters.

^1^Median (interquartile range).

^2^CRT and BCVA are expressed as mean (95% confidence interval).

### Optical coherence tomography biomarkers in eyes with ME-RVO

Different OCT biomarkers have been identified in patients with RVO. They included CRT and choroidal thickness; external limiting membrane (ELM) and ellipsoid zone (EZ) integrity; disorganization of retinal inner layers (DRIL); presence of subretinal fluid (SRF); and hyperreflective foci (HRF) (69, 74, 89–91). Moreover, in treatment-naïve eyes with ME secondary to BRVO and presence of SRF, there was a significant correlation between baseline EZ status and 12-months BCVA (91).

In addition, several signs of ischemia (i.e., prominent middle limiting membrane, paracentral acute middle maculopathy, and hyperreflectivity of inner retinal layers) have been identified as predictors of poor efficacy outcomes in eyes with RVO (89, 90, 92–97).

Few studies have evaluated the predictors of DEX-i outcomes in eyes with ME-RVO.

Arrigo et al. (67) found that older age, presence of diabetes mellitus, and greater baseline CRT were associated with a higher risk for repeat DEX-i injection. Moreover, regarding CRT, the median time to the second DEX-i in eyes with baseline CRT > 375 μm was significantly shorter (4.06 months) than in those eyes with baseline CRT < 375 μm (more than 50 months), *p* = 0.022 (69).

Additionally, in a retrospective study counting only 35 eyes with ME-RVO, Ding et al. (98) compared the effect of anti-VEGF and DEX-i. It has been shown a greater improvement of serous retinal detachment (SND) and a greater reduction in the number of HRF after DEX-i, however due to the small sample of the study and the methodology further evidence are required to draw conclusion. These findings may suggest that, as compared to anti-VEGF, anti-inflammatory therapy has a greater capacity to resolve serous retinal detachment and HRF in patients with ME-RVO.

Castro-Navarro et al. (74) assessed, in a retrospective study, potential OCT biomarkers associated with DEX-i visual and anatomic outcomes in eyes with ME-RVO (both BRVO and CRVO). According to the results of this study, in the univariate analysis the presence of 10–20 HRF at baseline (*p* = 0.0361) and the change in septum status (present at baseline versus absent at month-6) (*p* = 0.0014) were predictors of anatomic success; while baseline disrupted ELM and baseline BCVA (*p* = 0.0007) were predictors of achieving a BCVA ≥ 15 letters at month 6. Furthermore, the baseline BCVA was significantly associated with functional success in the multivariate analysis (*p* = 0.0057) (74).

Shi et al. (99) found that baseline LogMAR BCVA was positively correlated with SRF and macular thickness, while the number of HRF at baseline was negatively correlated with foveal thickness.

In summary, based on current evidence, different OCT and OCT-angiography (OCTA) biomarkers have been associated with both visual and anatomic outcomes in eyes with RVO. A better understanding of these biomarkers will help to provide an accurate prognosis and an individualized treatment selection in eyes with ME-RVO.

Table 4 shows the main OCT biomarkers identified in patients with RVO.

**TABLE 4:** Key optical coherence tomography (OCT) and optical coherence tomography angiography (OCTA) biomarkers in retinal vein occlusion (RVO).

Biomarker	Significance	Predictor of good response to steroids
**Optical coherence tomography**		
IRC	Indicate the presence of ME, particularly associated with vasogenic mechanisms.	No data
CST	Increased thickness is positively correlated with poorer visual outcomes.	Yes
DRIL	The morphological extent is associated with the severity of vision loss, reflecting the degree of cellular damage.	No data
ELM rupture	Associated with poorer visual prognosis.	No data
EZ disruption	Associated with poorer visual prognosis.	No data
HRF	Inflammatory biomarker correlated with poorer visual outcomes, likely resulting from damage to the photoreceptor layer.	Yes
SRF	Inflammatory biomarker commonly observed at initial presentation; however, it does not reliably predict long-term functional or anatomical outcomes.	Yes
**Optical coherence tomography angiography**		
FAZ	Elevated in both the superficial and deep capillary plexuses.	No Data
CV	Evident in both the superficial and deep capillary plexuses, positively correlating with an enlarged FAZ and increased CST, while negatively correlating with VA.	No Data
CFD	Reduced in the presence of ME-RVO.	No Data
CVI	Increase in the presence of ME-RVO.	No Data

IRC, intraretinal cysts; CST, intraretinal cysts; DRIL, disorganization of retinal inner layers; ELM, external limiting membrane; EZ, ellipsoid zone; HRF, hyperreflective foci; SRF, subretinal fluid; ME, macular edema; FAZ, foveal avascular zone; CV, collateral vessels; VA, visual acuity; CFD, choriocapillaris flow density; ME-RVO, macular edema secondary to retinal vein occlusion CVI, choroidal vascularity index. The information to build this table has been extracted from references (9, 14, 69, 74, 89–99, 126).

**FIGURE 3:**
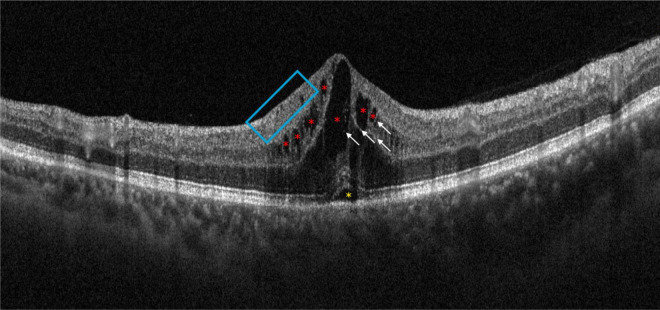
Optical coherence tomography (OCT) scan of patient with central retinal vein occlusion (CRVO). It is possible to observe some of the main morphological biomarkers: intraretinal cysts (red asterisks), subretinal fluid (yellow asterisk), some hyperreflective foci (white arrows), and disorganization of the retinal inner layers (blue box).

In addition, the Figure 3 shows different OCT biomarkers of an eye diagnosed with CRVO.

### Safety of DEX-i in eyes with retinal vein occlusion

DEX-i is a relatively safe procedure, although it is not free of adverse events (AEs). The AEs associated with DEX-i do not differ from those observed with other corticosteroids, including the onset or progression of cataract and the rise of intraocular pressure (IOP), which can be effectively managed with pharmacological or surgical intervention. Other Intravitreal injection related AEs less frequently reported are retinal detachment, vitreous hemorrhage, and endophthalmitis (25, 100, 101).

#### Cataract

The development of a new cataract or worsening of a preexisting cataract is a relatively common complication associated with DEX-i injection (25, 100, 101).

According to the information reported by the 5 RCTs included in the current study, cataract-related adverse events (CatRAEs) ranged between 0.9% (31) and 11.3% (37).

Among all the RWE studies evaluated, CatRAEs ranged between 0% (50, 72) in 6-months follow-up studies and 70.4% in a 36-months follow-up study (50).

As can be seen in Table 5 and as has been reported in the literature (25, 100, 101), the rate of CatRAEs depends, critically, on the duration of study follow-up. Indeed, it has been previously reported that the risk of cataract is higher in eyes receiving multiple DEX-i injections (101).

**TABLE 5:** Rate of patients treated with intravitreal dexamethasone implant (DEX-i) who reported any adverse event, cataract related adverse events, or an increase in intraocular pressure (IOP) during the study.

Study	References	Design	Eyes (n)	LOFU, months	Disease	Any AEs	CatRAEs	Increase IOP*
Li et al.	(34)	RCT	63 66	6	BRVO CRVO	69 (53.5%)	2 (0.9%)	38 (29.5%)
COMRADE-B	(35)	RCT	118	6	BRVO	74 (62.7%)	4 (3.8%)	6 (5.1%)^a^
COMO	(36)	RCT	154	12	BRVO	127 (3.0%)	13 (8.5%)	50 (32.7%)
COMRADE**	(37)	RCT	40 22	12	BRVO CRVO	38 (61.3%)	7 (11.2%)	14 (22.6)
Hussain et al.	(42)	R	10 12	6	BRVO CRVO	N.P.	6 (27.3%)	5 (22.7%)
Yuksel et al.	(43)	R	15	6	BRVO	N.P.	N.P.	6 (53.3%)
Lip et al.	(44)	R	28 9 29	12	BRVO HRVO CRVO	N.P.	12 (18%)^b^	19 (41%)
Mishra et al.	(46)	P	20	6	CRVO	N.P.	2 (10%)	5 (25%)
Yoon et al.	(47)	P	71	12	BRVO	35 (49%)	11 (16%)	25 (35%)
Garay-Aramburu et al.	(48)	R	10	60	BRVO CRVO	N.P.	3 (7.5%)	11 (27.5%)
Simsek et al.	(49)	P	40 31	4	BRVO CRVO	N.P.	6 (12.2%)	7 (17.5%)
Blanc et al.	(50)	R	21 29	36	BRVO CRVO	N.P.	31 (70.4%)	36 (54.5%)
Kaldırım et al.	(51)	R	20	6	BRVO	N.P.	1 (5%)	5 (25%)
Donati et al.	(52)	P	8 18	6	BRVO CRVO	N.P.	0 (0.0%)	4 (15.4%)
Hou et al.	(54)	R	32 40	6	BRVO CRVO	N.P.	18 (25%)	12 (16.7%)
Ozkaya et al.	(55)	R	41	24	BRVO	N.P.	12 (29.3%)	9 (22%)
Tufail et al.	(56)	P	385 257	24	BRVO CRVO	386 (57.2)	177 (26.2%)	138 (20.4)
Yucel et al.	(57)	R	24	6	CRVO	N.P.	7 (29.2%)	5 (20.8%)
Georgalas et al.	(59)	P	13 10	12	BRVO CRVO	N.P:	2 (8.7%)	5 (21.7%)
Teja et al.	(60)	R	28	6	RVO	N.P.	N.P.	10 (36%)
Bayat et al.	(61)	R	19	6	BRVO	N.P.	4 (21.1%)	4 (21.1%)
Dikel et al.	(62)	R	16	12	BRVO	N.P:	1 (6.3%)	3 (18.8%)
Nikula et al.	(63)	R	12 24	48 W	BRVO CRVO	13 (36.1%)	3 (8.3%)	5 (13.9%)
Wallsh et al.	(64)	R	90 59	10 y	BRVO CRVO	N.P.	26 (28.9%) 33 (55.9%)	N.P.
Erogul et al.	(66)	R	22 19	3	BRVO CRVO	N.P.	2 (9.1%)	N.P.
Arrigo et al.	(67)	R	135 178	7 y	BRVO CRVO	N.P.	N.P.	N.P.
Huang et al.	(69)	R	29	30	BRVO	N.P.	4 (13.8%)	7 (24.1%)
Wecker et al.	(70)	R	99	36	BRVO CRVO	N.P.	13 (13.1%)	N.P.^c^
Harb et al.	(71)	P	38	12	BRVO CRVO	N.P.	N.P.	8 (21.1%)
Kim et al.	(72)	P	554 146	6	BRVO CRVO	53 (7.6%)	1 (0.1%)	37 (5.3%)
Garay-Aramburu et al.	(73)	R	77 34	44	BRVO CRVO	N.P.	32.1%	16.8%
Castro-Navarro et al.	(74)	R	42 15	6	BRVO CRVO	N.P.	0 (0.0%)	0 (0.0%)
Jirarattanasopa et al.	(75)	R	12 2 14	6	BRVO HRVO CRVO	N.P.	5 (21.7%)	9 (20.5%)
Zhang et al.	(76)	R	6 2	5 y	BRVO CRVO	N.P.	1 (12.5%)	N.P.
Soliman et al.	(79)	R	862 688	24	BRVO CRVO	N.P.	100 (8.3%)	76 (11%)
Ozcift et al.	(80)	R	23	6	BRVO	N.P:	0 (0.0%)	3 (13%)
Darabuş et al.	(82)	P	15	6	BRVO	N.P.	N.P.	4 (26.7%)
Ayaz et al.^†^	(83)	R	91 45	24	BRVO CRVO	142 (30.2%)^†^	55 (21.7%)^ 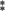 ^	97 (20.6) ^†^

*IOP increased was defined as either an elevation in IOP compared to baseline or an IOP > of a specific value or need to receive ocular hypotensive treatment.

**COMRADE Extension.

^†^Percentages calculated from the total of 470 eyes. total of 470 eyes.

^
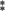
^Percentage calculated from 253 phakic eyes.

^a^Ocular hypertension.

^b^Cataract surgeries.

^c^Over 75% of patients at all observation time points did not require any IOP-lowering therapy during the entire follow-up period. AEs, adverse events; BRVO, branch retinal vein occlusion; CRVO, central retinal vein occlusion; CatRAE, cataract-related adverse events; IOP, intraocular pressure; NP, not provided; NA, not available.

The results of a multicenter study that evaluated data of 6,015 DEX-i injections in 2,736 eyes found that, among the phakic eyes, 576 (32.5%) eyes developed cataract requiring surgical intervention; whereas, 259 (14.6%) developed cataract that did not impact on VA and, therefore, did not require surgery (100).

According to the results of the United Kingdom Database Study (77), 8.3% (100/1,202) of the phakic eyes underwent cataract surgery in a median follow-up period of 16 months. At month-12, the accumulative probability of cataract surgery was 4%; while at month-24 such probability increased to 15% (79).

According to the current evidence, phakic patients should expect cataract progression with the need of cataract surgery within roughly 1 year after DEX-i injection (25, 34–38, 40–83, 100, 101).

#### Elevation of intraocular pressure

Elevation of intraocular pressure is probably the most frequently reported adverse event associated with the use of DEX-i (25, 34–83, 100–102).

However, it is extremely complex to establish comparisons between the different studies, since the definition of “Increased/elevated IOP” varies greatly between them (Table 5).

While some studies have reported adverse events related to increased IOP as a function of ocular hypotensive treatments administered during study follow-up, other papers reported IOP elevation > a specific value, and other ones defined “Increased IOP” as an IOP > 21 mmHg or > 25 mmHg (34–83).

Moreover, according to data from a study that included a total of 6,015 injections in 2,736 eyes of 1,441 patients only 0.5% of eyes who experienced IOP increased required glaucoma surgery (100).

The SAFODEX study evaluated the incidence of IOP related events in 421 eyes receiving one or more DEX-i (103). Among 1,000 DEX-i injected, the rise of IOP (defines as either at least 25 mmHg or an IOP elevation ≥ 10 mmHg from baseline) was recorded for 28.5% of injected eyes over a mean follow-up period of 16.8 months (range: 3–55 months). Ocular hypotensive drops were administered to 31% of eyes; while only three eyes, with preexisting glaucoma, required filtering surgery for managing IOP elevation (103). Factors significantly associated with a higher risk of IOP elevation were younger age, male sex, type 1 diabetes, preexisting glaucoma treated with 2 or 3 ocular hypotensive drops, and a history of RVO (103).

More recently, the SAFODEX-2 Study (104), evaluated the long-term incidence of IOP related events in 494 eyes treated with DEX-i. In particular, of 1,371 DEX-i injected eyes, 32.6% of injected eyes showed a rise of IOP (defined as reference 106), with a median follow-up of 29 months (range: 3 to 62.5 months) (104). Topical hypotensive therapy controlled successfully 97% of IOP elevations. Interestingly, there was not an accumulative effect after repeated DEX-i injection (i.e., incidence of a rise of IOP did not change regardless the number of DEX-i injected).

In addition, the International Ozurdex Study Group reported an incidence rate of IOP elevation (IOP > 25 mmHg) of 26.5% (727/2,736 eyes). The increase in IOP was successfully controlled with ocular hypotensive therapy in more than 90% of eyes, while only 0.5% of eyes required filtering surgery (100).

In summary, IOP elevation was more frequent in patients with a previous history of ocular hypertension/glaucoma and, in most cases, was successfully managed with ocular hypotensive therapy. Patients with preexisting ocular hypertension or glaucoma have to be always informed of this possible risk and should be followed with regular checks, to recognize these complications as soon as possible and to treat them appropriately (56, 83, 100, 102–104).

#### Neovascular glaucoma

One of the most serious complications secondary to ischemic CRVO is neovascular glaucoma (105) and it is related to the increase of VEGF and inflammation (11).

The imbalance between anti-angiogenic and VEGF factors is one of the causes of the new-vessels formation (106). In addition, different inflammatory molecules, including interleukin (IL)-6, IL-8, platelet-derived growth factor, and interferon-α (among others), have been involved in the onset of neovascular glaucoma (107).

To our knowledge, there is no evidence indicating that DEX-i increases the risk of neovascular glaucoma onset.

There is evidence that anti-VEGF agents can delay the onset of neovascular glaucoma, but not prevent it (11). Furthermore, evidence regarding the long-term effectiveness of anti-VEGF medications is uncertain (108). This means that acting simultaneously on VEGF and inflammation, as DEX-i acts (109), may be more effective when treating macular edema consequent to RVO without an increased risk of developing neovascular glaucoma.

Hayreh’s natural history studies reported that 12–33% of not ischemic CRVO become ischemic within 4 years (110). Nevertheless, GENEVA study found a significant reduction in retinal neovascularization from 2.6 to 0.7% at 6 months (23), suggesting that DEX-i might reduce the development of ischemic complications (23, 25).

#### Other adverse events

The long-term safety profile of DEX-i was assessed in a prospective and multicenter post marketing study, which involved 102 centers in France, Germany, Spain, and United Kingdom, that included patients with RVO (73.1% of them with a follow-up of 24-months) (56). The incidence rate of “adverse events of special interest” (other than cataract-related AE or increased IOP) was vitreous hemorrhage (3.7%); glaucoma (1.5%); retinal detachment (0.6%); vitreous detachment (0.6%); infectious endophthalmitis (0.1%); implant misplacement (0.1%); and implant dislocation (0.1%) (56).

In a retrospective study that evaluated the safety profile of 925 injections of DEX-i (for various retinal diseases, such as diabetic macular edema, RVO, postoperative cystoid macular edema, etc.), 1 (0.3%) patient required glaucoma surgery for controlling IOP; 3 (0.8%) patients had an anterior chamber dislocation of the implant; and 1 (0.3%) had a sterile endophthalmitis that required pars-plana vitrectomy (83).

Finally, in a multicenter and retrospective study that included 6,015 injections in 2,736 eyes of 1,441 patients, who underwent treatment with DEX-i for treating different retinal diseases (including RVO, diabetic macular edema, post-surgical cystoid macular edema, and uveitis) the incidence of ocular AEs was endophthalmitis (0.07%), retinal detachment (0.03%), vitreous hemorrhage (0.03%) (100).

In summary, according to the results of these studies, the incidence rate of these AEs was low.

### Is ocular perfusion/ischemia relevant for selecting the treatment of choice?

Pathophysiological factors, such as overexpression of different cytokines, VEGF, hypoxia, and inflammation have been associated with ME-RVO (11, 20, 111).

There is broad evidence supporting that both anti-VEGF and DEX-i target these factors and, therefore, are the treatment of choice of ME-RVO (14, 22, 86, 87).

Despite these treatment options have provided good visual and anatomic outcomes, in many cases, they were time-limited and the anatomic analysis were focused on structural changes of ME-RVO (14, 22, 86, 87).

In addition, the sole resolution of ME-RVO may not be sufficient for achieving good visual outcome, since macular capillary network and the perifoveal capillary arcade alterations have been associated with impaired visual outcomes (112–114).

OCT-angiography has gained relevance as the microvascular abnormalities, especially vessel density in the deep retinal vascular plexus, the foveal avascular zone, and of areas with no capillary perfusion, have been associated with clinical outcomes in eyes with RVO (90, 92–97, 113, 114).

According to the results of a five-year, retrospective, and real-world study DEX-i achieved better retinal perfusion than anti-VEGF in eyes with ME-RVO (76).

Indeed, eyes treated with DEX-i had smaller foveal avascular zone (FAZ) areas and greater FAZ circularity index (i.e., better regularity of the FAZ region in the DEX-i treated eyes) and a significantly greater perfusion density (*p* = 0.001) in both superficial and deep capillary plexuses when compared to eyes treated with anti-VEGF (76). Moreover, the ratio of perfusion density was greater in the eyes treated with DEX-i than in those treated with anti-VEGF which suggested that DEX-i provided a higher degree of perfusion advantage in the deep capillary plexus (76).

This finding may be explained by the quick resolution of ME by DEX-i (115). Collateral circulation and residual capillary reperfusion can improve ischemia associated with ME-RVO (116, 117). It might be hypothesized that the faster ME-RVO resolved, the better retinal perfusion and clinical outcomes were showed (76).

OCTA has shown to be a useful tool for accurately assess the dynamics of macular and retinal microvasculature, which may help to choose the most appropriate treatment for these patients.

In summary, better vascular perfusion and a smaller FAZ area in both the superficial and deep capillary plexuses were significantly associated with better VA after treatment of ME-BRVO. Therefore, preserving retinal perfusion, especially in the deep capillary plexus, seems to be crucial for better VA in eyes with ME-BRVO (113). Since DEX-i improves vascular perfusion, particularly in the deep capillary plexus, it might be the treatment of choice in patients with retinal ischemia (76, 98, 113).

In this context, the analysis carried out by Gok et al. (118) on retrobulbar hemodynamics provided also insight into the effect of single DEX-i on ocular blood flow velocities in patients with RVO. The results of this study showed no significant effect on the ocular blood flow velocities and changes registered were in acceptable limits. Though they demonstrated the reversibility effect of DEX-i on ocular microcirculation, attributing these effects to DEX-i properties on stabilizing the vascular wall and decreasing the leakage inhibiting the VEGF and inflammation-mediated vasodilatory effects (118).

### Current strategies for the management of macular edema secondary to RVO

In 2019, the “*Guidelines for the Management of Retinal Vein Occlusion by the European Society of Retina Specialists (EURETINA)*” were published (14). They suggested a series of updated recommendations about the clinical management of RVO, based on scientific evidence and the opinion/experience of renowned experts in this field.

Briefly, panretinal laser photocoagulation was considered the treatment of choice for neovascular complications associated with RVO, including retinal and disk neovascularization secondary to RVO as well as iris neovascularization (14).

Anti-VEGF (ranibizumab 0.5 mg/aflibercept 2 mg) were considered as the first-line therapy, since both have been shown to provide anatomic and functional improvements in ME-RVO. However, both treatments require multiple injections and a monthly follow-up period for at least 1 year (14).

Because inflammation is part of the pathophysiological process of ME-RVO, the use of DEX-i for its treatment is indicated. There are evidences suggesting that, in addition to inhibit the expression of proinflammatory and inflammatory molecules, corticosteroids also decrease the expression of VEGF gene and its metabolic pathways (11, 14, 21). However, despite the evidence supporting the good anatomic and visual outcomes of DEX-i, this Guidelines recommended its use as a second-choice treatment (14). DEX-i may be considered as a first line therapy for patients presenting a recent history of major cardiovascular events and in those who are not able to follow the monthly injections (and/or monitoring) regimen during the first 6 months of therapy (14).

More updated guidelines have been released in 2022, “*The Royal College of Ophthalmologists RVO Guidelines*” (86, 87). Their main purpose was to provide evidence-based, clinical guidance for the appropriate management of different features of RVO, based on current scientific evidence and on consensus opinion of a representative expert panel with a special interest/expertise in this disease (86, 87).

For both center-involving EM secondary to BRVO and secondary to CRVO, these Guidelines recommended to start intravitreal therapy with either intravitreal injections of anti-VEGF or DEX-i. The choice of treatment will be based on clinician and patient’s features, cardiovascular comorbidities, treatment frequency, risk of IOP rise and cataract formation (86, 87).

In eyes with ischemic CRVO or those with disk or retinal neovascularization and/or iris neovascularization, panretinal photocoagulation would be indicated, either alone or in combination with intravitreal therapy (84, 85).

The combined analysis of the Phase 3 controlled BALATON and COMINO trials demonstrated that both visual acuity and anatomical outcomes with faricimab were non-inferior to those observed with aflibercept. In terms of safety, the incidence of ocular adverse events was comparable between patients treated with faricimab and those receiving aflibercept (119).

Recent scientific evidence has demonstrated the potential of combining DEX implant therapy with anti-VEGF treatment for managing ME-RVO, including cases of ME-CRVO (120) and ME-BRVO (121). Both studies reported that this combined approach led to sustained improvements in BCVA in RVO patients, while reducing the need for frequent re-injections and minimizing complications (120, 121).

Since the publication of the EURETINA Guidelines (14), much evidence has been suggested regarding the use of DEX-i in patients with ME-RVO.

This has led to a change in the current therapeutic algorithm, in which the first-line treatment must be decided based on multiple factors, such as the patient’s comorbidities, the ability of the clinician and patient to comply with the treatment regimen and/ or visits, the lens status (phakic versus pseudophakic), ophthalmological history (for example, ocular hypertension or glaucoma), etc (14, 86, 87).

### Does make sense to switch from anti-VEGF to DEX-i?

As far as we know, there is limited evidence supporting that in eyes who did not properly respond to an anti-VEGF agent, switching to another anti-VEGF agent may be effective (122).

Georgalas et al. (59), in a 12-month prospective, nonrandomized, and interventional study evaluated the effect of DEX-i in eyes with persistent ME secondary to either BRVO or CRVO, who underwent treatment with at least anti-VEGF injections. According to the results of this study, therapeutic peak effect of DEX-i on BCVA was achieved at 4 months in BRVO (0.3 logMAR improvement from baseline) and at 2 months in CRVO (0.4 logMAR improvement) (59).

The anatomic and visual outcomes of a single DEX-i, in eyes with ME-RVO who were resistant to at least three-monthly injections of ranibizumab, were assessed in a retrospective study (123). As compared to baseline, DEX-i significantly improved mean BCVA at month-2 (*p* = 0.03) and month-3 (*p* = 0.003) and significantly reduced CRT at months 2,3, and 6 (*p* = 0.00003, *p* = 0.00003, and *p* = 0.03, respectively) (123).

In a retrospective case series, switching to DEX-i in eyes who did not properly respond to anti-VEGF therapy (defined as ≤ +5 ETDRS letter gain and ≤ 20% reduction in central subfield thickness following ≥ 6 consecutive anti-VEGF injections) resulted in significant visual acuity improvement (+6 letters, 95% CI +2.2 to +9.1 letters, *p* < 0.01) at 30 days (124).

Additionally, a retrospective systematic review and metanalysis published in 2022, which included 99 eyes from 99 patients, evaluated the anatomic and visual outcomes of DEX-i in eyes with refractory ME-RVO previously treated with anti-VEGF (125). The pooled results demonstrated a significant mean BCVA improvement of -0.23 logMAR (*p* = 0.004) at month-2, -0.20 logMAR (*p* = 0.027) at month-3, and -0.09 logMAR (*p* = 0.021) at month-6 after DEX-i injection (118). Regarding baseline CRT, it was significantly reduced by 241.89 μm at month-2, 222.61 μm at month-3, and 90.49 μm (*p* < 0.001 each, respectively) (125).

Finally, a recent review primarily analyzed the key pathophysiological and clinical factors that could favor transitioning from anti-VEGF therapy to intravitreal DEX-i in RVO (126). According to this paper, this switch is mainly determined by parameters such as BCVA and CRT, typically after a minimum of three anti-VEGF injections. However, the role of OCT biomarkers related to inflammation, which could help identify patients who may benefit more from DEX-i therapy, is often overlooked (126).Unfortunately, this review could not offer definitive guidance on the optimal timing for switching to DEX implant in RVO patients. The clinical data available is highly variable, and specific imaging biomarkers that could assist in making this therapeutic decision have yet to be identified (126).

In summary, in eyes with ME-RVO who did not adequately respond to anti-VEGF therapy, switching to DEX-i provided better anatomic and visual outcomes (59, 123–126).

## Discussion

Depending on the anatomical site of occurrence, RVO can be classified as BRVO, CRVO (it can be subdivided in ischemic and non-ischemic), and HRVO, which is an intermediary between CRVO and BRVO (9, 127).

The pathogenesis of RVO has not been thoroughly understood, since many other diseases interact with it, including cardiovascular diseases, systemic diseases, and glaucoma (11, 14).

Macular edema, the main responsible of vision loss associated with RVO (either BRVO or CRVO), is characterized by increased intraluminal pressure, vascular endothelial damage, and impaired blood–retina barrier (BRB) that results in leakage, and precipitation of lipoproteins (15). In addition, the release of proinflammatory and inflammatory mediators by the damaged tissue exacerbates the pathogenesis of ME (20, 21, 111).

Different factors, including endothelial dysfunction, inflammation, increased hydrostatic venous pressure, and increased vascular permeability have been associated with the onset of ME-RVO (15, 18, 128, 129).

Upregulation of VEGF and local inflammation within the eye have been identified as one of the leading mediators of RVO pathogenesis and symptoms (20, 21, 111).

Retinal vein occlusion causes inflammation because of retinal ischemia and hemorrhage. Consequently, there is evidence that upregulation of inflammatory factors, including VEGF, VEGF receptor 2 (VEGFR-2), intercellular adhesion molecule 1 (ICAM-1), IL-6, and MCP-1, or downregulation of anti-inflammatory factors, such as pigment epithelium-derived factor (PEDF), and a subsequent increase in leukocyte-endothelial interactions contribute to breakdown of the BRB (15, 20, 21). Additionally, activation of VEGF receptors 1 and 2 increases vascular permeability and the expression of different inflammatory molecules (15, 20, 21). This results in leukocyte chemotaxis and adhesion to the vascular endothelium leading to increased ischemia and inflammation (15, 20, 21).

There is growing evidence to support the relationship between the inflammatory response and RVO-ME (20, 21, 130, 131). Inflammatory markers present in the aqueous and vitreous, including VEGF, IL-6, IL-8, TNF-α, etc., are regarded as significant factors in the pathogenesis of ME-RVO (15, 131).

Different RCTs have demonstrated the efficacy and safety on anti-VEGF in patients with ME-RVO (both BRVO and CRVO) (132–140). However, the use of anti-VEGF agents has been linked to the need for frequent injections. Although the precise number of injections required to initiate treatment remains unclear, studies suggest that six monthly injections yield better outcomes compared to four monthly injections (132–140).

Gale et al. (88), in a real-world study evaluating data from 5,661 treatment-naive patients, found that those patients treated with anti-VEGF received, on average, 5.1 injections at 12-months (as compared to 1.5 DEX-i).

Modi et al. (141) found that among 3,099 eyes with ME secondary to BRVO, 1,197 (38.6%) received ≤ 6 injections (mean injections: 4.6, range: 2–6) and 1,902 (61.4%) received ≥ 7 injections through 1 year (mean injections: 8.8, range: 7–13). Moreover, they reported that mean VA improvement from baseline was significantly lower in the eyes receiving ≤ 6-injections than in those treated with ≥ 7-injections at year 1 (10.4 vs. 13.9; *p* < 0.001) (141).

Finally, the results of a meta-analysis found that at 12 months, the mean number of anti-VEGF injections was 8.1 (95% CI: 7.4–8.7) (142).

Despite the transformative impact of anti-VEGF agents on the clinical management of ME-RVO, challenges persist (142). The need for several injections leads to a significant treatment burden for patients and financial cost for the healthcare system (143, 144).

Additionally, although anti-VEGF have demonstrated to be effective for treating ME-RVO, visual acuity subsequently decreases after initial improvements (132–140). Moreover, this loss of peak vision occurred in almost all patients, it is greater in patients with poor visual outcome, but also occurring in patients with good visual outcome (145).

Therefore, other intravitreal treatments, such as DEX-i, have emerged to minimize injection frequency while maintaining good outcomes (23, 25, 34–83, 144, 146, 147).

The scientific analyzed evidence (see Tables 1, [T2]2) has demonstrated that DEX-i significantly improved visual function and retinal macular anatomical morphology in eyes with ME-RVO.

Furthermore, the results of a meta-analysis that included a total of 1,248 patients with ME-RVO showed that DEX-i was more effective than anti-VEGF in terms of BCVA improvement and CRT reduction but slightly less safe than anti-VEGF therapy (147).

The clinical outcomes of DEX-i have been noted as early as day 7, achieving its peak of action at day 60. The mean duration of its anatomic and visual outcomes was approximately 4 months (23, 25, 34–83, 146–148).

The longer duration of its effects is associated with a fewer number of injections and medical visits, which significantly reduces the economic burden (14, 86–88, 144).

In this regard treating patients who are naïve to previous therapies may offer an opportunity to achieve a more durable treatment response. These patients may have a less complicated disease history, allowing for better predictability of the treatment’s long-term efficacy. In naïve patients, there might be an opportunity to establish an effective treatment plan from the outset, potentially reducing the need for frequent injections over time (49, 50, 63, 66, 69, 71, 72, 74).

Therefore, the potential benefits of an early switching to DEX-i treatment or initiating treatment in naïve patients align with efforts to optimize visual outcomes, provide long-lasting durability, and reduce the burden of injections in this patient population (49, 50, 63, 66, 69, 71, 72, 74).

It is essential to note that while these potential advantages are promising, individual patient responses can vary, and the decision to switch to or initiate DEX-i treatment should be based on a thorough assessment of the patient’s specific clinical characteristics and needs.

Nowadays side effects related to DEX-i are well known and large experience as thought physician how to manage it. In those cases, who experience an IOP increase, topical ocular hypotensive treatment is enough in most patients (15, 25, 34–83, 88, 100–102). Regarding cataract related AEs, they are mainly related to the number of injections. According to the results of the GENEVA trial, there was no difference compared to sham / also in the UK database 4% of cataract was developed after 1 year (15, 25, 34–83, 88, 100–102).

In addition to the elevation of IOP and the risk of cataract development/progression, other AEs related to the use of DEX-i have been identified, although their incidence rate is very low (15, 25, 34–83, 88, 100–102).

DEX-i is generally injected after 4 months in a *pro-re-nata* (PRN) regimen (86, 87). According to *The Royal College of Ophthalmologists RVO Guidelines* (86, 87), both anti-VEGF and DEX-i may be considered as treatment of choice for patients with ME secondary to either CRVO or BRVO, depending on clinician and patient preferences, and considering frequency of treatment/visits, risk of IOP elevation, and lens status.

Additionally, considering that poor vascular perfusion was associated with worse visual outcomes (113), understanding whether intravitreal agents can improve retinal perfusion, particularly in the deep capillary plexus, may help to determine whether these agents may of benefit as first line treatments. It has been suggested that, compared with anti-VEGF, DEX-i can improve retinal perfusion, especially in the deep capillary plexus, with a longer-lasting effect over time in patients with retinal vein occlusion macular edema (76), however further confirmatory trials are needed.

## Conclusion

Based on the current evidence, DEX-i significantly improved BCVA and reduced CRT in eyes with ME-RVO (both BRVO and CRVO) quickly and for a long period.

The main adverse events associated with the use of DEX-i are IOP elevation (medically controlled in most cases) and cataract development/progression, that remain manageable complications.

Selecting the correct treatment for the right patient with ME-RVO is the key to achieve good visual and anatomical outcomes. In pursuit of this goal, the identification of biomarkers capable of accurately predicting the prognosis of the disease and the adoption of a patient-centered approach, guided by their clinical characteristics, are crucial.

## Data Availability

Data sharing was not applicable to this article as no datasets were generated or analyzed during the current study.

## Footnotes

1
http://www.prisma-statement.org/

## References

[1] Wong T Scott I. Clinical practice. Retinal-vein occlusion. *N Engl J Med.* (2010). 363(22):2135–44. 10.1056/NEJMcp1003934 21105795

[2] Hayreh S. Prevalent misconceptions about acute retinal vascular occlusive disorders. *Prog Retin Eye Res.* (2005) 24(4):493–519. 10.1016/j.preteyeres.2004.12.001 15845346

[3] O’Mahoney P Wong D Ray J. Retinal vein occlusion and traditional risk factors for atherosclerosis. *Arch Ophthalmol.* (2008) 126(5):692–9. 10.1001/archopht.126.5.692 18474782

[4] Klein R Klein B Moss S Meuer S. The epidemiology of retinal vein occlusion: The Beaver Dam Eye Study. *Trans Am Ophthalmol Soc.* (2000) 98:133–41. 11190017 PMC1298220

[5] Rogers S McIntosh R Cheung N Lim L Wang J Mitchell P International eye disease consortium. The prevalence of retinal vein occlusion: Pooled data from population studies from the United States, Europe, Asia, and Australia. *Ophthalmology.* (2010) 117(2):313–9.e1. 10.1016/j.ophtha.2009.07.017 20022117 PMC2945292

[6] Song P Xu Y Zha M Zhang Y Rudan I. Global epidemiology of retinal vein occlusion: A systematic review and meta-analysis of prevalence, incidence, and risk factors. *J Glob Health.* (2019) 9(1):010427. 10.7189/jogh.09.010427 31131101 PMC6513508

[7] EURETINA, *Retinal Diseases in Europe: Whitebook on Prevalence, Incidence and Healthcare Needs.* (2017). Available online at: https://euretina.org/resource/euretina-whitebook-on-prevalence-incidence-and-healthcare-needs-for-retinal-diseases-in-europe/ (accessed October 3, 2024).

[8] Wong T Larsen E Klein R Mitchell P Couper D Klein B Cardiovascular risk factors for retinal vein occlusion and arteriolar emboli: The atherosclerosis risk in communities & cardiovascular health studies. *Ophthalmology.* (2005) 112(4):540–7. 10.1016/j.ophtha.2004.10.039 15808241

[9] Rehak M Wiedemann P. Retinal vein thrombosis: Pathogenesis and management. *J Thromb Haemost.* (2010) 8(9):1886–94. 10.1111/j.1538-7836.2010.03909.x 20492457

[10] Yau J Lee P Wong T Best J Jenkins A. Retinal vein occlusion: An approach to diagnosis, systemic risk factors and management. *Intern Med J.* (2008) 38(12):904–10. 10.1111/j.1445-5994.2008.01720.x 19120547

[11] Tang Y Cheng Y Wang S Wang Y Liu P Wu H. Review: The development of risk factors and cytokines in retinal vein occlusion. *Front Med.* (2022) 9:910600. 10.3389/fmed.2022.910600 35783660 PMC9240302

[12] Trovato Battagliola E Pacella F Malvasi M Scalinci S Turchetti P Pacella E Risk factors in central retinal vein occlusion: A multi-center case-control study conducted on the Italian population: Demographic, environmental, systemic, and ocular factors that increase the risk for major thrombotic events in the retinal venous system. *Eur J Ophthalmol.* (2022) 32(5):2801–9. 10.1177/11206721211064469 34854784

[13] Li J Wei J Wang Y Bair H Hsu S Lin CJ. Retinal vascular occlusion and Covid-19 diagnosis: A multicenter population-based study. *Retina.* (2024) 44(2):345–52. 10.1097/IAE.0000000000003952 37824812

[14] Schmidt-Erfurth U Garcia-Arumi J Gerendas B Midena E Sivaprasad S Tadayoni R Guidelines for the management of retinal vein occlusion by the european society of retina specialists (EURETINA). *Ophthalmologica.* (2019) 242(3):123–62. 10.1159/000502041 31412332

[15] Daruich A Matet A Moulin A Kowalczuk L Nicolas M Sellam A Mechanisms of macular edema: Beyond the surface. *Prog Retin Eye Res.* (2018) 63:20–68. 10.1016/j.preteyeres.2017.10.006 29126927

[16] Witmer A Vrensen G Van Noorden C Schlingemann R. Vascular endothelial growth factors and angiogenesis in eye disease. *Prog Retin Eye Res.* (2003) 22(1):1–29. 10.1016/S1350-9462(02)00043-5 12597922

[17] Wautier J Wautier M. Vascular permeability in diseases. *Int J Mol Sci.* (2022) 23(7):3645. 10.3390/ijms23073645 35409010 PMC8998843

[18] Ehlers J Fekrat S. Retinal vein occlusion: Beyond the acute event. *Surv Ophthalmol.* (2011) 56(4):281–99. 10.1016/j.survophthal.2010.11.006 21601903

[19] Ascaso F Huerva V Grzybowski A. The role of inflammation in the pathogenesis of macular edema secondary to retinal vascular diseases. *Mediators Inflamm.* (2014) 2014:432685. 10.1155/2014/432685 25152567 PMC4134827

[20] Noma H Yasuda K Shimura M. Cytokines and the pathogenesis of macular edema in branch retinal vein occlusion. *J Ophthalmol.* (2019) 2019:5185128. 10.1155/2019/5185128 31191997 PMC6525954

[21] Noma H Yasuda K Shimura M. Cytokines and pathogenesis of central retinal vein occlusion. *J Clin Med.* (2020) 9(11):3457. 10.3390/jcm9113457 33121094 PMC7692731

[22] Chatziralli I Nicholson L Sivaprasad S Hykin P. Intravitreal steroid and anti-vascular endothelial growth agents for the management of retinal vein occlusion: Evidence from randomized trials. *Expert Opin Biol Ther.* (2015) 15(12):1685–97. 10.1517/14712598.2015.1086744 26358547

[23] Haller J Bandello F Belfort R Jr. Blumenkranz M Gillies M Heier J Randomized, sham-controlled trial of dexamethasone intravitreal implant in patients with macular edema due to retinal vein occlusion. *Ophthalmology.* (2010) 117(6):1134–46.e3. 10.1016/j.ophtha.2010.03.032 20417567

[24] Ip M Scott I VanVeldhuisen P Oden N Blodi B Fisher M Study Research Group. A randomized trial comparing the efficacy and safety of intravitreal triamcinolone with observation to treat vision loss associated with macular edema secondary to central retinal vein occlusion: The Standard Care vs Corticosteroid for Retinal Vein Occlusion (SCORE) study report 5. *Arch Ophthalmol.* (2009) 127(9):1101–14. 10.1001/archophthalmol.2009.234 19752419 PMC2872173

[25] Haller J Bandello F Belfort R Jr. Blumenkranz M Gillies M Heier J Dexamethasone intravitreal implant in patients with macular edema related to branch or central retinal vein occlusion twelve-month study results. *Ophthalmology.* (2011) 118(12):2453–60. 10.1016/j.ophtha.2011.05.014 21764136

[26] Liberati A Altman D Tetzlaff J Mulrow C Gøtzsche P Ioannidis J The prisma statement for reporting systematic reviews and meta-analyses of studies that evaluate healthcare interventions: Explanation and elaboration. *BMJ.* (2009) 339:b2700. 10.1136/bmj.b2700 19622552 PMC2714672

[27] Petticrew M Anderson L Elder R Grimshaw J Hopkins D Hahn R Complex interventions and their implications for systematic reviews: A pragmatic approach. *J Clin Epidemiol.* (2013) 66(11):1209–14. 10.1016/j.jclinepi.2013.06.004 23953085

[28] Donnelly C Boyd I Campbell P Craig C Vallance P Walport M Four principles to make evidence synthesis more useful for policy. *Nature.* (2018) 558(7710):361–4. 10.1038/d41586-018-05414-4 29925978

[29] Campbell M McKenzie J Sowden A Katikireddi S Brennan S Ellis S Synthesis without meta-analysis (SWiM) in systematic reviews: Reporting guideline. *BMJ.* (2020) 368:l6890. 10.1136/bmj.l6890 31948937 PMC7190266

[30] Valentine J Wilson S Rindskopf D Lau T Tanner-Smith E Yeide M Synthesizing evidence in public policy contexts: The challenge of synthesis when there are only a few studies. *Eval Rev.* (2017) 41(1):3–26. 10.1177/0193841X16674421 27780906

[31] Campbell M Katikireddi S Sowden A Thomson H. Lack of transparency in reporting narrative synthesis of quantitative data: A methodological assessment of systematic reviews. *J Clin Epidemiol.* (2019) 105:1–9. 10.1016/j.jclinepi.2018.08.019 30196129 PMC6327109

[32] Page M McKenzie J Bossuyt P Boutron I Hoffmann T Mulrow C The prisma 2020 statement: An updated guideline for reporting systematic reviews. *BMJ.* (2021) 372:n71. 10.1136/bmj.n71 33782057 PMC8005924

[33] Yilmaz M Citirik M Rahmanlar H Alkan A Gursoz H. Evaluation of off-label anti-vascular endothelial growth factor and steroid implant medication uses in macular edema due to retinal vein occlusion in Turkey. *J Clin Pharm Ther.* (2022) 47(12):2101–6. 10.1111/jcpt.13757 36543255

[34] Li X Wang N Liang X Xu G Li X Jiao J Safety and efficacy of dexamethasone intravitreal implant for treatment of macular edema secondary to retinal vein occlusion in Chinese patients: Randomized, sham-controlled, multicenter study. *Graefes Arch Clin Exp Ophthalmol.* (2018) 256(1):59–69. 10.1007/s00417-017-3831-6 29119239 PMC5748421

[35] Hattenbach L Feltgen N Bertelmann T Schmitz-Valckenberg S Berk H Eter N Head-to-head comparison of ranibizumab PRN versus single-dose dexamethasone for branch retinal vein occlusion (COMRADE-B). *Acta Ophthalmol.* (2018) 96(1):e10–8. 10.1111/aos.13381 28251811

[36] Bandello F Augustin A Tufail A Leaback RA. 12-month, multicenter, parallel group comparison of dexamethasone intravitreal implant versus ranibizumab in branch retinal vein occlusion. *Eur J Ophthalmol.* (2018) 28(6):697–705. 10.1177/1120672117750058 29631435 PMC6210573

[37] Feltgen N Hattenbach L Bertelmann T Callizo J Rehak M Wolf A Comparison of ranibizumab versus dexamethasone for macular oedema following retinal vein occlusion: 1-year results of the COMRADE extension study. *Acta Ophthalmol.* (2018) 96(8):e933–41. 10.1111/aos.13770 29855153

[38] Kumar P Sharma Y Chandra P Azad R Meshram G. Comparison of the safety and efficacy of intravitreal ranibizumab with or without laser photocoagulation versus dexamethasone intravitreal implant with or without laser photocoagulation for macular edema secondary to branch retinal vein occlusion. *Folia Med.* (2019) 61(2):240–8. 10.2478/folmed-2018-0081 31301668

[39] Hoerauf H Feltgen N Weiss C Paulus E Schmitz-Valckenberg S Pielen A Clinical efficacy and safety of ranibizumab versus dexamethasone for central retinal vein occlusion (COMRADE C): A European label study. *Am J Ophthalmol.* (2016) 169:258–67. 10.1016/j.ajo.2016.04.020 27163237

[40] Winterhalter S Eckert A Vom Brocke G Schneider A Pohlmann D Pilger D Real-life clinical data for dexamethasone and ranibizumab in the treatment of branch or central retinal vein occlusion over a period of six months. *Graefes Arch Clin Exp Ophthalmol.* (2018) 256(2):267–79. 10.1007/s00417-017-3852-1 29185099

[41] Altunel O Göktaş A Duru N Özköse A Arifoðlu H Atağ M. The effect of age on dexamethasone intravitreal implant (Ozurdex§) response in macular edema secondary to branch retinal vein occlusion. *Semin Ophthalmol.* (2018) 33(2):179–84. 27599562 10.1080/08820538.2016.1188128

[42] Hussain R Ciulla T Ciulla L Sink B Harris A. Efficacy of dexamethasone intravitreal implant for refractory macular edema caused by retinal vein occlusion. *Retin Cases Brief Rep.* (2018) 12(4):294–9. 10.1097/ICB.0000000000000496 27984545

[43] Yuksel B Karti O Celik O Kerci S Kusbeci T. Low frequency ranibizumab versus dexamethasone implant for macular oedema secondary to branch retinal vein occlusion. *Clin Exp Optom.* (2018) 101(1):116–22. 10.1111/cxo.12586 28922697

[44] Lip P Cikatricis P Sarmad A Damato E Chavan R Mitra A Efficacy and timing of adjunctive therapy in the anti-VEGF treatment regimen for macular oedema in retinal vein occlusion: 12-month real-world result. *Eye.* (2018) 32(3):537–45. 10.1038/eye.2017.230 29099501 PMC5848273

[45] Bulut M Çallı Ü Akçay G Kıvrak U Bulut K Özertürk Y. Effects of dexamethasone implant on multifocal electroretinography in central retinal vein occlusion. *J Ophthalmic Vis Res.* (2018) 13(1):23–8. 10.4103/jovr.jovr_118_16 29403585 PMC5782451

[46] Mishra S Gupta A Patyal S Kumar S Raji K Singh A Intravitreal dexamethasone implant versus triamcinolone acetonide for macular oedema of central retinal vein occlusion: Quantifying efficacy and safety. *Int J Retina Vitreous.* (2018) 4:13. 10.1186/s40942-018-0114-2 29632703 PMC5883339

[47] Yoon Y Kim J Lee J Kim I Kang S Yu H Dexamethasone intravitreal implant for early treatment and retreatment of macular edema related to branch retinal vein occlusion: The multicenter cobalt study. *Ophthalmologica.* (2018) 240(2):81–9. 10.1159/000487547 29642062 PMC6106142

[48] Garay-Aramburu G Gómez-Moreno Á. A 5-year follow-up study of the treatment of macular edema due to retinal vein occlusion using dexamethasone intravitreal implants. *J Ocul Pharmacol Ther.* (2018) 34(6):436–41. 10.1089/jop.2017.0148 29708803

[49] Simsek M Citirik M Ozates S Ozkoyuncu D. The efficacy of intravitreal dexamethasone implant as the first-line treatment for retinal vein occlusion-related macular edema in a real-life scenario. *Indian J Ophthalmol.* (2018) 66(6):831–6. 10.4103/ijo.IJO_1259_17 29785994 PMC5989508

[50] Blanc J Deschasse C Kodjikian L Dot C Bron A Creuzot-Garcher C. Safety and long-term efficacy of repeated dexamethasone intravitreal implants for the treatment of cystoid macular edema secondary to retinal vein occlusion with or without a switch to anti-VEGF agents: A 3-year experience. *Graefes Arch Clin Exp Ophthalmol.* (2018) 256(8):1441–8. 10.1007/s00417-018-4016-7 29855706

[51] Kaldırım H Yazgan SA. comparison of three different intravitreal treatment modalities of macular edema due to branch retinal vein occlusion. *Int Ophthalmol.* (2018) 38(4):1549–58. 10.1007/s10792-017-0618-z 28646440

[52] Donati S Gandolfi C Caprani S Cattaneo J Premoli L Azzolini C. Evaluation of the effectiveness of treatment with dexamethasone intravitreal implant in cystoid macular edema secondary to retinal vein occlusion. *Biomed Res Int.* (2018) 2018:3095961. 10.1155/2018/3095961 30175123 PMC6098881

[53] Niro A Sborgia G Sborgia A Sborgia L Furino C Recchimurzo N Analysis of morphologic and functional outcomes in macular edema due to central retinal vein occlusion treated with intravitreal dexamethasone implant. *J Ophthalmol.* (2018) 2018:5604632. 10.1155/2018/5604632 30271628 PMC6151240

[54] Houben I De Zaeytijd J Deghislage C Frost N Nijs I Van Calster J. Efficacy of multiple dexamethasone intravitreal implants for refractory retinal vein occlusion-related macular edema and effect of prior vitrectomy. *J Ocul Pharmacol Ther.* (2018) 34(10):710–9. 10.1089/jop.2018.0012 30403557

[55] Ozkaya A Tarakcioglu H Tanir I. Ranibizumab versus dexamethasone implant in macular edema secondary to branch retinal vein occlusion: Two-year outcomes. *Optom Vis Sci.* (2018) 95(12):1149–54. 10.1097/OPX.0000000000001306 30451802

[56] Tufail A Lightman S Kamal A Pleyer U Paniagua N Dot C Post-marketing surveillance study of the safety of dexamethasone intravitreal implant in patients with retinal vein occlusion or noninfectious posterior segment uveitis. *Clin Ophthalmol.* (2018) 12:2519–34. 10.2147/OPTH.S181256 30584271 PMC6287662

[57] Yucel O Birinci H Sullu Y. The short-term efficacy of intravitreal ranibizumab, aflibercept and dexamethasone implant in the treatment of macular edema due to non-ischemic central retinal vein occlusion. *Int Ophthalmol.* (2019) 39(4):891–901. 10.1007/s10792-018-0890-6 29550932

[58] Eris E Perente I Erdogan G Seymen Z Sucu M Ozkaya A Clinical use of dexamethasone implants in resistant macular edema secondary to branch retinal vascular occlusion compared with intravitreal anti-vascular endothelial growth factor. *Beyoglu Eye J.* (2019) 4(1):11–6. 10.14744/bej.2019.63835 35187425 PMC8842035

[59] Georgalas L Tservakis I Kiskira E Petrou P Papaconstantinou D Kanakis M. Efficacy and safety of dexamethasone intravitreal implant in patients with retinal vein occlusion resistant to anti-VEGF therapy: A 12-month prospective study. *Cutan Ocul Toxicol.* (2019) 38(4):330–7. 10.1080/15569527.2019.1614020 31060385

[60] Teja S Sawatzky L Wiens T Maberley D Ma P. Ozurdex for refractory macular edema secondary to diabetes, vein occlusion, uveitis and pseudophakia. *Can J Ophthalmol.* (2019) 54(5):540–7. 10.1016/j.jcjo.2018.12.005 31564342

[61] Bayat A Bölükbasi S Erden B Cakir A Ozturan S Tayfur M A real-life study: Intravitreal aflibercept, ranibizumab and dexamethasone for macular edema secondary to branch retinal vein occlusion. *Beyoglu Eye J.* (2019) 4(3):131–5. 10.14744/bej.2019.18291 35187448 PMC8842062

[62] Dikel N Yumusak E Gokcinar N. Long-term results of ranibizumab, dexamethasone implant, and triamcinolone in macular edema due to branch retinal vein occlusion. *Beyoglu Eye J.* (2019) 4(3):141–8. 35187450 10.14744/bej.2019.40412 PMC8842065

[63] Nicula C Nicula D Rednik A Bulboaca A Crişan O. Morphological and functional outcomes after intravitreal dexamethasone injection for macular edema in patients with central vein occlusion at 48-week follow-up. *J Ophthalmol.* (2020) 2020:6830148. 10.1155/2020/6830148 32104595 PMC7036098

[64] Sever O Cakir A Horozoglu F. A comparison of the effect of intravitreal dexamethasone implant on macular and branch retinal vein occlusion. A real life experience. *Eur J Ophthalmol.* (2020) 30(5):1106–11. 10.1177/1120672120934982 32530706

[65] Wallsh J Luths C Kil H Gallemore R. Initial ten years of experience with the intravitreal dexamethasone implant: A retrospective chart review. *Clin Ophthalmol.* (2020) 14:3097–108. 10.2147/OPTH.S264559 33116361 PMC7548337

[66] Erogul O Yozgat Z Sabaner M Dogan M Gobeka H. The effect of intravitreal dexamethasone implant on central foveal thickness and choroidal thickness in retinal vein occlusion. *Niger J Clin Pract.* (2021) 24(1):121–6. 10.4103/njcp.njcp_87_20 33473037

[67] Arrigo A Crepaldi A Viganò C Aragona E Lattanzio R Scalia G Real-life management of central and branch retinal vein occlusion: A seven-year follow-up study. *Thromb Haemost.* (2021) 121(10):1361–6. 10.1055/s-0041-1725197 33706397

[68] de Salles M Epstein D. Real-life study of the use of anti-VEGF therapy versus dexamethasone implant for treatment of macular edema in retinal vein occlusion. *Graefes Arch Clin Exp Ophthalmol.* (2021) 259(9):2653–60. 10.1007/s00417-021-05146-8 33738625 PMC8380569

[69] Huang Y Lin C Chen H Tien P Lai C Hsia N Risk factors for repeated dexamethasone intravitreal implant therapy for macular edema due to treatment-naïve branch retinal vein occlusion. *BMC Ophthalmol.* (2021) 21(1):142. 10.1186/s12886-021-01904-8 33743610 PMC7981849

[70] Wecker T Grundel B Grundel M Bründer M Trick S Lange C Real-life medium term follow-up data for intravitreal dexamethasone implant in retinal vein occlusion. *Sci Rep.* (2021) 11(1):8303. 10.1038/s41598-021-87467-6 33859243 PMC8050279

[71] Harb W Chidiac G Harb G. Outcomes of combination therapy using aflibercept and dexamethasone intravitreal implant versus dexamethasone monotherapy for macular edema secondary to retinal vein occlusion. *Middle East Afr J Ophthalmol.* (2021) 28(1):18–22. 10.4103/meajo.MEAJO_297_19 34321818 PMC8270014

[72] Kim M Choi J Lee H Woo S Korea Ozurdex Post-Marketing Surveillance Study Group Dexamethasone intravitreal implant for the treatment of macular edema following retinal vein occlusion: Post hoc analysis of post-marketing surveillance data in the real-world setting in Korea. *Clin Ophthalmol.* (2021) 15:3623–36. 10.2147/OPTH.S302014 34475750 PMC8407780

[73] Garay-Aramburu G Gómez-Moreno Á Urcola A. Short-term effectiveness prognostic factors after dexamethasone intravitreal implant in macular edema due to retinal vein occlusion. *Eur J Ophthalmol.* (2022) 32(3):1671–9. 10.1177/11206721211032520 34254547

[74] Castro-Navarro V Monferrer-Adsuara C Navarro-Palop C Montero-Hernández J Cervera-Taulet E. Optical coherence tomography biomarkers in patients with macular edema secondary to retinal vein occlusion treated with dexamethasone implant. *BMC Ophthalmol.* (2022) 22(1):191. 10.1186/s12886-022-02415-w 35473615 PMC9040324

[75] Jirarattanasopa P Jiranoppasakdawong S Ratanasukon M Bhurayanontachai P Dangboon W. Results of intravitreal dexamethasone implant (Ozurdex§) for retinal vascular diseases with macular edema: An observational study of real-life situations. *Medicine.* (2022) 101(27):e29807. 10.1097/MD.0000000000029807 35801769 PMC9259138

[76] Zhang M Liu Y Song M Yu Y Ruan S Zheng K Intravitreal dexamethasone implant has better retinal perfusion than anti-vascular endothelial growth factor treatment for macular edema secondary to retinal vein occlusion: A five-year real-world study. *Ophthalmic Res.* (2023) 66(1):247–58. 10.1159/000527447 36215955

[77] Moreno-López M de-Arriba-Palomero P de-Arriba-Palomero F Ituruburu F Dompablo E Negrete F. Prospective evaluation of the effectiveness of combined treatment of macular edema secondary to retinal vein occlusion with intravitreal bevacizumab and dexamethasone implants. *Arq Bras Oftalmol.* (2023) 86(3):255–62. 10.5935/0004-2749.20230040 35319651 PMC11826545

[78] Horozoglu F Sener H Polat O Temizyurek O Evereklioglu C. Predictive impact of optical coherence tomography biomarkers in anti-vascular endothelial growth factor resistant macular edema treated with dexamethasone implant. *Photodiagnosis Photodyn Ther.* (2023) 42:103167. 10.1016/j.pdpdt.2022.103167 36261095

[79] Soliman M Zarranz-Ventura J Chakravarthy U McKibbin M Brand C Menon G United Kingdom ozurdex in retinal vein occlusion electronic medical records (UK Ozurdex RVO EMR) users group. United Kingdom database study of intravitreal dexamethasone implant (ozurdex) for macular edema related to retinal vein occlusion. *Retina.* (2023) 43(4):679–87. 10.1097/IAE.0000000000003698 36729561

[80] Ozcift S Aydin E Egrilmez, Koc F Eris E. Comparison of intravitreal dexamethasone implant and intravitreal ranibizumab efficacy in younger patients with branch retinal vein occlusion. *Beyoglu Eye J.* (2023) 8(1):21–5. 36911221 10.14744/bej.2023.42243 PMC9993420

[81] Yu-Chuan Kang E Shao S Chang K Garg S Lin T Chen K Real-world effectiveness of intravitreal dexamethasone implants - comparison between eyes eligible and ineligible for clinical trials and their associated outcomes. *Biomed J.* (2023) 47:100607. 10.1016/j.bj.2023.100607 37196877 PMC10826172

[82] Darabuş D Munteanu M Preda M Karancsi O Şuşă MC. The impact of intraocular treatment on visual acuity of patients diagnosed with branch retinal vein occlusions. *Healthcare.* (2023) 11(10):1414. 10.3390/healthcare11101414 37239696 PMC10217981

[83] Ayaz Y Erkan Pota Ç Başol Ý Doğan ME Türkoğlu Şen EB Ünal M. Anterior segment complications after dexamethasone implantations:real world data. *Int Ophthalmol.* (2023) 43(11):4279–87. 10.1007/s10792-023-02838-4 37707746

[84] Ming S Xie K Yang M He H Li Y Lei B. Comparison of intravitreal dexamethasone implant and anti-VEGF drugs in the treatment of retinal vein occlusion-induced oedema: A meta-analysis and systematic review. *BMJ Open.* (2020) 10(6):e032128. 10.1136/bmjopen-2019-032128 32595145 PMC7322510

[85] Coscas G Loewenstein A Augustin A Bandello F Battaglia Parodi M Lanzetta P Management of retinal vein occlusion–consensus document. *Ophthalmologica.* (2011) 226(1):4–28. 10.1159/000327391 21577038

[86] The Royal College of Ophthalmologist. *Retinal Vein Occlusion (RVO) Guidelines.* (2022). Available online at: Retinal-Vein-Occlusion-Guidelines-2022.pdf (rcophth.ac.uk) (accessed September 19, 2023).

[87] Nicholson L Talks S Amoaku W Talks K Sivaprasad S. Retinal vein occlusion (RVO) guideline: Executive summary. *Eye.* (2022) 36(5):909–12. 10.1038/s41433-022-02007-4 35301458 PMC9046155

[88] Gale R Pikoula M Lee A Denaxas S Egan C Tufail A Real world evidence on 5661 patients treated for macular oedema secondary to branch retinal vein occlusion with intravitreal anti-vascular endothelial growth factor, intravitreal dexamethasone or macular laser. *Br J Ophthalmol.* (2021) 105(4):549–54. 10.1136/bjophthalmol-2020-315836 32532760 PMC8005793

[89] Yin S Cui Y Jiao W Zhao B. Potential prognostic indicators for patients with retinal vein occlusion. *Front Med.* (2022) 9:839082. 10.3389/fmed.2022.839082 35692537 PMC9174432

[90] Siedlecki J Hattenbach L Feltgen N Priglinger S. Biomarker in der therapie venöser retinaler Gefäßverschlüsse [Biomarkers in the treatment of retinal vein occlusion]. *Ophthalmologie.* (2022) 119(11):1111–20. 10.1007/s00347-022-01732-1 36201041

[91] Sasajima H Zako M Murotani K Ishida H Ueta Y Tachi N Visual prognostic factors in eyes with subretinal fluid associated with branch retinal vein occlusion. *J Clin Med.* (2023) 12(8):2909. 10.3390/jcm12082909 37109246 PMC10146202

[92] Ghasemi Falavarjani K Iafe N Hubschman J Tsui I Sadda S Sarraf D. Optical coherence tomography angiography analysis of the foveal avascular zone and macular vessel density after anti-vegf therapy in eyes with diabetic macular edema and retinal vein occlusion. *Invest Ophthalmol Vis Sci.* (2017) 58(1):30–4. 10.1167/iovs.16-20579 28114569

[93] Kadomoto S Muraoka Y Ooto S Miwa Y Iida Y Suzuma K Evaluation of macular ischemia in eyes with branch retinal vein occlusion: An optical coherence tomography angiography study. *Retina.* (2018) 38(2):272–82. 10.1097/IAE.0000000000001541 28221256

[94] Ghashut R Muraoka Y Ooto S Iida Y Miwa Y Suzuma K Evaluation of macular ischemia in eyes with central retinal vein occlusion: An optical coherence tomography angiography study. *Retina.* (2018) 38(8):1571–80. 10.1097/IAE.0000000000001749 28671896

[95] Yeung L Wu W Chuang L Wang N Lai C. Novel optical coherence tomography angiography biomarker in branch retinal vein occlusion macular edema. *Retina.* (2019) 39(10):1906–16. 10.1097/IAE.0000000000002264 30028408

[96] Huang Y Wang I Lin C Lai C Hsia N Chen H Comparison of choroidal thickness, foveal avascular zone, and macular capillary density in macular edema secondary to branch retinal vein occlusion treated with ranibizumab or aflibercept-a prospective study. *Medicina.* (2022) 58(4):540. 10.3390/medicina58040540 35454378 PMC9031043

[97] Frederiksen K Pedersen F Vergmann A Yang D Laugesen C Vestergaard J Predictive value of retinal oximetry, optical coherence tomography angiography and microperimetry in patients with treatment-naïve branch retinal vein occlusion. *Int J Retina Vitreous.* (2023) 9(1):38. 10.1186/s40942-023-00468-7 37381014 PMC10308602

[98] Ding X Hu Y Yu H Li Q. Changes of optical coherence tomography biomarkers in macular edema secondary to retinal vein occlusion after anti-VEGF and anti-inflammatory therapies. *Drug Des Devel Ther.* (2022) 16:717–25. 10.2147/DDDT.S351683 35313554 PMC8934115

[99] Shi K Sun X Zhang J. Optical coherence tomography angiography characteristics serve as retinal vein occlusion therapeutic biomarkers for dexamethasone intravitreal implant. *Dis Markers.* (2021) 2021:3510036. 10.1155/2021/3510036 34691285 PMC8528596

[100] Rajesh B Zarranz-Ventura J Fung A Busch C Sahoo N Rodriguez-Valdes P for international Ozurdex study group. safety of 6000 intravitreal dexamethasone implants. *Br J Ophthalmol.* (2020) 104(1):39–46. 10.1136/bjophthalmol-2019-313991 31040132

[101] Reid G Sahota D Sarhan M. Observed complications from dexamethasone intravitreal implant for the treatment of macular edema in retinal vein occlusion over 3 treatment rounds. *Retina.* (2015) 35(8):1647–55. 10.1097/IAE.0000000000000524 25932545

[102] Mansoori T Shiva S Balakrishna N. Ocular hypertension and its predictor after a single Ozurdex implant: One-year follow-up in Asian Indian eyes. *Oman J Ophthalmol.* (2022) 15(3):299–303. 10.4103/ojo.ojo_326_21 36760962 PMC9905915

[103] Malclès A Dot C Voirin N Vié A Agard É Bellocq D Safety of intravitreal dexamethasone implant (Ozurdex): The safodex study. Incidence and risk factors of ocular hypertension. *Retina.* (2017) 37(7):1352–9. 10.1097/IAE.0000000000001369 27768641

[104] Rezkallah A Mathis T Abukhashabah A Voirin N Malclès A Agard É. Long-term incidence and risk factors of ocular hypertension following dexamethasone-implant injections: The safodex-2 study. *Retina.* (2021) 41(7):1438–45. 10.1097/IAE.0000000000003080 33315814

[105] Brown G Magargal L Schachat A Shah H. Neovascular glaucoma. Etiologic considerations. *Ophthalmology.* (1984) 91(4):315–20. 10.1016/S0161-6420(84)34293-2 6201791

[106] Boyd S Zachary I Chakravarthy U Allen G Wisdom G Cree I Correlation of increased vascular endothelial growth factor with neovascularization and permeability in ischemic central vein occlusion. *Arch Ophthalmol.* (2002) 120(12):1644–50. 10.1001/archopht.120.12.1644 12470137

[107] Tam E Golchet P Yung M DeCroos F Spirn M Lehmann-Clarke L Ischemic central retinal vein occlusion in the anti-vascular endothelial growth factor era. *Retina.* (2018) 38(2):292–8. 10.1097/IAE.0000000000001546 28207609

[108] Simha A Aziz K Braganza A Abraham L Samuel P Lindsley K. Anti-vascular endothelial growth factor for neovascular glaucoma. *Cochrane Database Syst Rev.* (2020) 2(2):CD007920. 10.1002/14651858.CD007920.pub3 32027392 PMC7003996

[109] Sarao V Veritti D Boscia F Lanzetta P. Intravitreal steroids for the treatment of retinal diseases. *ScientificWorldJournal.* (2014) 2014:989501. 10.1155/2014/989501 24526927 PMC3910383

[110] Hayreh S. Management of central retinal vein occlusion. *Ophthalmologica.* (2003) 217(3):167–88. 10.1159/000068980 12660480

[111] Haydinger C Ferreira L Williams K Smith J. Mechanisms of macular edema. *Front Med.* (2023) 10:1128811. 10.3389/fmed.2023.1128811 36960343 PMC10027768

[112] Murakami T Tsujikawa A Miyamoto K Sakamoto A Ogino K Muraoka Y Disrupted foveal photoreceptors after combined cystoid spaces and retinal detachment in branch vein occlusion treated with bevacizumab. *Retina.* (2012) 32(9):1853–61. 10.1097/IAE.0b013e31824b824c 22481482

[113] Wakabayashi T Sato T Hara-Ueno C Fukushima Y Sayanagi K Shiraki N Retinal microvasculature and visual acuity in eyes with branch retinal vein occlusion: Imaging analysis by optical coherence tomography angiography. *Invest Ophthalmol Vis Sci.* (2017) 58(4):2087–94. 10.1167/iovs.16-21208 28388705

[114] Javed A Khanna A Palmer E Wilde C Zaman A Orr G Optical coherence tomography angiography: A review of the current literature. *J Int Med Res.* (2023) 51(7):3000605231187933. 10.1177/03000605231187933 37498178 PMC10387790

[115] Chiquet C Dupuy C Bron A Aptel F Straub M Isaico R Intravitreal dexamethasone implant versus anti-VEGF injection for treatment-naïve patients with retinal vein occlusion and macular edema: A 12-month follow-up study. *Graefes Arch Clin Exp Ophthalmol.* (2015) 253(12):2095–102. 10.1007/s00417-015-2947-9 25673251

[116] Kamei M Matsumura N Suzuki M Sakimoto S Sakaguchi H Nishida K. Reperfusion of large ischemic areas associated with central retinal vein occlusion: A potential novel treatment with activated protein C. *JAMA Ophthalmol.* (2014) 132(3):361–2. 10.1001/jamaophthalmol.2013.6334 24626830

[117] Wada T Song Y Oomae T Sogawa K Yoshioka T Nakabayashi S Longitudinal changes in retinal blood flow in a feline retinal vein occlusion model as measured by doppler optical coherence tomography and optical coherence tomography angiography. *Invest Ophthalmol Vis Sci.* (2020) 61(2):34. 10.1167/iovs.61.2.34 32084269 PMC7326600

[118] Gok M Altas H Kapti H. The impact of intravitreal dexamethasone implant (Ozurdex§) on retrobulbar hemodynamics in patients with diabetic macular edema and retinal vein occlusions. *Cutan Ocul Toxicol.* (2019) 38(3):240–8. 10.1080/15569527.2019.1582061 30773934

[119] Tadayoni R Paris L Danzig C Abreu F Khanani A Brittain C Efficacy and safety of faricimab for macular edema due to retinal vein occlusion: 24-week results from the Balaton and comino trials. *Ophthalmology*. (2024) 131(8):950–60. 10.1016/j.ophtha.2024.01.029 38280653

[120] Cai X Zhao J Dang Y. Combination therapy with Anti-VEGF and intravitreal dexamethasone implant for macular edema secondary to retinal vein occlusion. *Curr Eye Res.* (2024) 49(8):872–8. 10.1080/02713683.2024.2343055 38639040

[121] Meng L Yang M Jiang X Li Y Han X. Comparing ranibizumab, dexamethasone implant, and combined therapy for macular edema secondary to branch retinal vein occlusion: A clinical trial. *Int Ophthalmol.* (2024) 44(1):262. 10.1007/s10792-024-03158-x 38913192

[122] Ip M Oden N Scott I VanVeldhuisen P Blodi B Ghuman T Month 12 outcomes after treatment change at month 6 among poor responders to aflibercept or bevacizumab in eyes with macular edema secondary to central or hemiretinal vein occlusion: A secondary analysis of the score2 study. *JAMA Ophthalmol.* (2019) 137(3):281–7. 10.1001/jamaophthalmol.2018.6111 30589922 PMC6439712

[123] Manousaridis K Peter S Mennel S. Outcome of intravitreal dexamethasone implant for the treatment of ranibizumab-resistant macular edema secondary to retinal vein occlusion. *Int Ophthalmol.* (2017) 37(1):47–53. 10.1007/s10792-016-0226-3 27043320

[124] Yap T Husein S Miralles de Imperial-Ollero JA Colizzi B Cordeiro MF Younis S. The efficacy of dexamethasone implants following anti-VEGF failure for macular oedema in retinal vein occlusion. *Eur J Ophthalmol.* (2021) 31(6):3214–22. 10.1177/1120672120978355 33356550

[125] Yuan Q Gao Y Liu Y Xu H Wang T Zhang M. Efficacy of single-dose intravitreal dexamethasone implantation for retinal vein occlusion patients with refractory macular edema: A systematic review and meta-analysis. *Front Pharmacol.* (2022) 13:951666. 10.3389/fphar.2022.951666 36249811 PMC9554209

[126] Vitiello L Lixi F Coppola A Abbinante G Gagliardi V Salerno G Intravitreal dexamethasone implant switch after anti-VEGF treatment in patients affected by retinal vein occlusion: A review of the literature. *J Clin Med*. (2024) 13(17):5006. 10.3390/jcm13175006 39274219 PMC11395781

[127] Ho M Liu D Lam D Jonas JB. Retinal vein occlusions, from BT. *Retina.* (2016) 36(3):432–48. 10.1097/IAE.0000000000000843 26716954

[128] Karia N. Retinal vein occlusion: Pathophysiology and treatment options. *Clin Ophthalmol.* (2010) 4:809–16. 10.2147/OPTH.S7631 20689798 PMC2915868

[129] Ip M Hendrick A. Retinal vein occlusion review. *Asia Pac J Ophthalmol.* (2018) 7(1):40–5. 10.22608/APO.2017163442 29280368

[130] Yoshimura T Sonoda K Sugahara M Mochizuki Y Enaida H Oshima Y Comprehensive analysis of inflammatory immune mediators in vitreoretinal diseases. *PLoS One.* (2009) 4(12):e8158. 10.1371/journal.pone.0008158 19997642 PMC2780733

[131] Wang B Zhang X Chen H Koh A Zhao C Chen YA. Review of intraocular biomolecules in retinal vein occlusion: Toward potential biomarkers for companion diagnostics. *Front Pharmacol.* (2022) 13:859951. 10.3389/fphar.2022.859951 35559255 PMC9086509

[132] Campochiaro P Heier J Feiner L Gray S Saroj N Rundle A Ranibizumab for macular edema following branch retinal vein occlusion: Six-month primary end point results of a phase III study. *Ophthalmology.* (2010) 117(6):1102–12.e1. 10.1016/j.ophtha.2010.02.021 20398941

[133] Brown D Campochiaro P Bhisitkul R Ho A Gray S Saroj N Sustained benefits from ranibizumab for macular edema following branch retinal vein occlusion: 12-month outcomes of a phase III study. *Ophthalmology.* (2011) 118(8):1594–602. 10.1016/j.ophtha.2011.02.022 21684606

[134] Campochiaro P Clark W Boyer D Heier J Brown D Vitti R Intravitreal aflibercept for macular edema following branch retinal vein occlusion: The 24-week results of the VIBRANT study. *Ophthalmology.* (2015) 122(3):538–44. 10.1016/j.ophtha.2014.08.031 25315663

[135] Clark W Boyer D Heier J Brown D Haller J Vitti R Intravitreal aflibercept for macular edema following branch retinal vein occlusion: 52-week results of the VIBRANT Study. *Ophthalmology.* (2016) 123(2):330–6. 10.1016/j.ophtha.2015.09.035 26522708

[136] Campochiaro P Brown D Awh C Lee S Gray S Saroj N Sustained benefits from ranibizumab for macular edema following central retinal vein occlusion: Twelve-month outcomes of a phase III study. *Ophthalmology.* (2011) 118(10):2041–9. 10.1016/j.ophtha.2011.02.038 21715011

[137] Brown D Heier J Clark W Boyer D Vitti R Berliner A Intravitreal aflibercept injection for macular edema secondary to central retinal vein occlusion: 1-year results from the phase 3 COPERNICUS study. *Am J Ophthalmol.* (2013) 155(3), 429–37.e7. 10.1016/j.ajo.2012.09.026 23218699

[138] Larsen M Waldstein S Boscia F Gerding H Monés J Tadayoni R Individualized ranibizumab regimen driven by stabilization criteria for central retinal vein occlusion: Twelve-month results of the CRYSTAL study. *Ophthalmology.* (2016) 123(5):1101–11. 10.1016/j.ophtha.2016.01.011 26896124

[139] Scott I VanVeldhuisen P Ip M Blodi B Oden N Awh C Effect of bevacizumab vs aflibercept on visual acuity among patients with macular edema due to central retinal vein occlusion: The SCORE2 randomized clinical trial. *JAMA.* (2017) 317(20):2072–87. 10.1001/jama.2017.4568 28492910 PMC5710547

[140] Hykin P Prevost A Vasconcelos J Murphy C Kelly J Ramu J Clinical effectiveness of intravitreal therapy with ranibizumab vs aflibercept vs bevacizumab for macular edema secondary to central retinal vein occlusion: A randomized clinical trial. *JAMA Ophthalmol.* (2019) 137(11):1256–64. 10.1001/jamaophthalmol.2019.3305 31465100 PMC6865295

[141] Modi Y Goduni L Moini H Gibson A Boucher N Lucas G Anti-vascular endothelial growth factor dosing frequency and visual outcomes in macular oedema following branch retinal vein occlusion. *Eye.* (2023) 37(16):3423–8. 10.1038/s41433-023-02527-7 37156863 PMC10630437

[142] Nanji K Khan M Khalid M Xie J Sarohia G Phillips M Treat-and-extend regimens of anti-vascular endothelial growth factor therapy for retinal vein occlusions: A systematic review and meta-analysis. *Acta Ophthalmol.* (2022) 100(6):e1199–208. 10.1111/aos.15068 34845830

[143] Comparison of Age-related Macular Degeneration Treatments Trials (CATT) Research Group, Maguire MG Martin DF Ying GS Jaffe GJ Daniel E Five-year outcomes with anti-vascular endothelial growth factor treatment of neovascular age-related macular degeneration: The comparison of age-related macular degeneration treatments trials. *Ophthalmology.* (2016) 123(8):1751–61. 10.1016/j.ophtha.2016.03.045 27156698 PMC4958614

[144] Sivaprasad S Oyetunde S. Impact of injection therapy on retinal patients with diabetic macular edema or retinal vein occlusion. *Clin Ophthalmol.* (2016) 10:939–46. 10.2147/OPTH.S100168 27307696 PMC4888735

[145] Iftikhar M Mir T Hafiz G Zimmer-Galler I Scott A Solomon S Loss of peak vision in retinal vein occlusion patients treated for macular edema. *Am J Ophthalmol.* (2019) 205:17–26. 10.1016/j.ajo.2019.03.029 30954469 PMC10019499

[146] Qiu X Hu X Qin Y Ma J Liu Q Qin L Comparison of intravitreal aflibercept and dexamethasone implant in the treatment of macular edema associated with diabetic retinopathy or retinal vein occlusion: A meta-analysis and systematic review. *Int J Ophthalmol.* (2022) 15(9):1511–9. 36124196 10.18240/ijo.2022.09.15 PMC9453398

[147] Xiaodong L Xuejun X. The efficacy and safety of dexamethasone intravitreal implant for diabetic macular edema and macular edema secondary to retinal vein occlusion: A meta-analysis of randomized controlled trials. *J Ophthalmol.* (2022) 2022:4007002. 10.1155/2022/4007002 35982771 PMC9381227

[148] Garweg J Zandi S. Retinal vein occlusion and the use of a dexamethasone intravitreal implant (Ozurdex§) in its treatment. *Graefes Arch Clin Exp Ophthalmol.* (2016) 254(7):1257–65. 10.1007/s00417-016-3350-x 27178087 PMC4917582

[149] Munk M Ceklic L Stillenmunkes R Chaudhary V Waheed N Chhablani J Integrated assessment of OCT, multimodal imaging, and cytokine markers for predicting treatment responses in retinal vein occlusion associated macular edema: A comparative review of Anti-VEGF and Steroid therapies. *Diagnostics*. (2024) 14(17):1983. 10.3390/diagnostics14171983 39272767 PMC11394301

